# A Raf-like protein kinase BHP mediates blue light-dependent stomatal opening

**DOI:** 10.1038/srep45586

**Published:** 2017-03-30

**Authors:** Maki Hayashi, Shin-ichiro Inoue, Yoshihisa Ueno, Toshinori Kinoshita

**Affiliations:** 1 Division of Biological Science, Graduate School of Science, Nagoya University, Chikusa, Nagoya 464-8602, Japan; 2 Institute of Transformative Bio-Molecules (WPI-ITbM), Nagoya University, Chikusa, Nagoya 464-8602, Japan

## Abstract

Stomata in the plant epidermis open in response to blue light and affect photosynthesis and plant growth by regulating CO_2_ uptake and transpiration. In stomatal guard cells under blue light, plasma membrane H^+^-ATPase is phosphorylated and activated via blue light-receptor phototropins and a signaling mediator BLUS1, and H^+^-ATPase activation drives stomatal opening. However, details of the signaling between phototropins and H^+^-ATPase remain largely unknown. In this study, through a screening of specific inhibitors for the blue light-dependent H^+^-ATPase phosphorylation in guard cells, we identified a Raf-like protein kinase, BLUE LIGHT-DEPENDENT H^+^-ATPASE PHOSPHORYLATION (BHP). Guard cells in the *bhp* mutant showed impairments of stomatal opening and H^+^-ATPase phosphorylation in response to blue light. BHP is abundantly expressed in the cytosol of guard cells and interacts with BLUS1 both *in vitro* and *in vivo*. Based on these results, BHP is a novel signaling mediator in blue light-dependent stomatal opening, likely downstream of BLUS1.

Stomata in the plant epidermis play critical roles in the regulation of photosynthesis and transpiration by promoting CO_2_ uptake and O_2_ and H_2_O release. A stomatal unit is composed of a pair of guard cells; stomatal pore opening and closing are controlled by guard cell-volume increases and decreases, respectively. Blue light in the sunshine opens stomata through guard cell swelling, and a single guard cell contains all of the signaling components for stomatal opening^1^,^2^. Blue light is perceived by the blue light-receptor protein kinase phototropins (phot1 and phot2) in guard cells and activates the phots to initiate signaling. Blue light-activated phot kinases induce activation of the plasma membrane H^+^-ATPase through phosphorylation of the C-terminal Thr^3^,^4^,^5^,^6^,^7^. The activated H^+^-ATPase establishes an electrochemical gradient of H^+^ and induces hyperpolarization of the guard cell plasma membrane^8^. The gradient is used for K^+^ uptakes into guard cells, which allows for swelling of the cells and stomatal opening^2^,^9^. Thus, C-terminal phosphorylation and subsequent activation of the guard cell H^+^-ATPase by blue light creates the driving force for K^+^ uptake and are crucial steps in stomatal opening^2^,^3^. However, detailed signaling between phots activation and H^+^-ATPase phosphorylation remains to be elucidated.

The plant plasma membrane H^+^-ATPase belongs to the P-type ATPase family, and the regulation mechanism of the enzyme is well-understood. In a steady state, activity of the H^+^-ATPase is maintained to a low level when the catalytic domain is inhibited by the C-terminal autoinhibitory region^10^,^11^,^12^. Phosphorylation of the penultimate Thr in this enzyme permits binding of 14-3-3 proteins to the phosphorylated C-terminus, and the binding induces a conformational change in the H^+^-ATPase that unhinges the autoinhibitory region from the catalytic domain, leading to H^+^-ATPase activation^3^,^13^,^14^,^15^. Very recently, a type 2 C protein phosphatase clade D (PP2C-D) was shown to function as a negative regulator of the H^+^-ATPase by direct dephosphorylation of the phosphorylated Thr in the C-terminus^16^. However, the protein kinases that directly phosphorylate the Thr and activate the H^+^-ATPase have not been identified.

During stomatal opening, two signaling components, BLUE LIGHT SIGNALING1 (BLUS1) and type 1 protein phosphatase (PP1), act as positive regulators between the phots and the H^+^-ATPase^17^,^18^. BLUS1 is directly bound and phosphorylated by phot kinases, and the phosphorylation is required for downstream signaling. Based on the results of pharmacological experiments, PP1 is thought to act downstream of BLUS1 and upstream of the H^+^-ATPase phosphorylation; however, the detailed relationship between PP1 and the other signaling components is largely unknown. In addition, there is an unidentified protein kinase that directly phosphorylates the H^+^-ATPase, as described above^7^. It has been shown that several kinase inhibitors suppress the blue light-dependent H^+^-ATPase activation and stomatal opening in Vicia and Arabidopsis guard cells^19^,^20^,^21^,^22^,^23^. Thus, protein phosphorylation events may play crucial roles in the blue light-dependent H^+^-ATPase phosphorylation, and there may be unknown protein kinases involved in the signaling, including the H^+^-ATPase kinase.

In this study, we screened the kinase inhibitors that suppress the blue light-dependent phosphorylation of the H^+^-ATPase in Arabidopsis guard cells. Consequently, we identified a Raf-like protein kinase, BHP, as a novel signaling-mediator for blue light-dependent phosphorylation of the H^+^-ATPase and stomatal opening. Furthermore, we found that BHP interacts with BLUS1, which is an early blue light-signaling component.

## Results

### Selection of inhibitors for the blue light-dependent phosphorylation of H^+^-ATPase and stomatal opening

**Figure 1:**
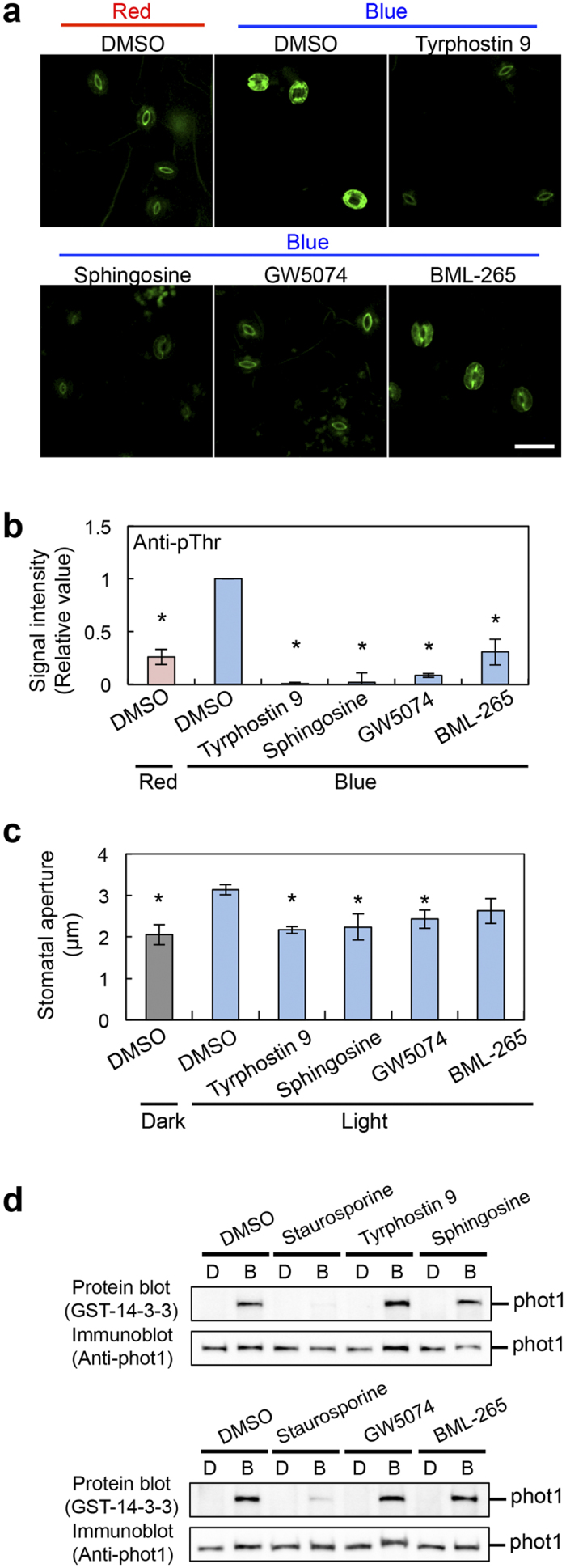
Selection of protein-kinase inhibitors that suppress blue light-dependent phosphorylation of guard cell H^+^-ATPase and stomatal opening. (**a**,**b**) Effects of four inhibitors on blue light-dependent H^+^-ATPase phosphorylation in Arabidopsis guard cells. Immunohistochemical detection of the phosphorylated H^+^-ATPase was performed using anti-pThr antibody in the leaf epidermis^23^. The fluorescent images (**a**) and relative intensities of the fluorescent signals (**b**) are shown. Epidermal fragments from dark-adapted plants were illuminated with red light (50 μmol m^−2^ s^−1^) for 20 min (Red), after which blue light was superimposed on the red right (10 μmol m^−2^ s^−1^) for 2.5 min (Blue). Each kinase inhibitor was administered at 50 μM to the epidermis under red light illumination. DMSO was used as a solvent control. The signal intensity was expressed as the ratio of the signal from the red light with DMSO or blue light with each inhibitor to that from blue light with DMSO. Scale bar represents 50 μm. Data indicate means ± SD (*n = *3) with measurement of 30 stomata in each sample. *indicates values that statistically differ from blue light sample without inhibitor (Student’s *t* test; *p < 0.01). (**c**) Effects of the four inhibitors on blue light-dependent stomatal opening. The epidermal fragments were pre-treated with each inhibitor at 10 μM for 20 min in the dark, and then illuminated with or without mixed light (red light at 50 μmol m^−2^ s^−1^ and blue light at 10 μmol m^−2^ s^−1^) for 3 h. Values indicate means ± SD (*n = *3); measurement of 30 stomata in each experiment. * indicates values that statistically differ from the light sample without inhibitor (Student’s *t* test; *p < 0.01). (**d**) Effects of the four inhibitors on phot1 activity. Blue light-dependent autophosphorylation of phot1 was determined by protein blotting using GST-14-3-3 protein (GF14ϕ) as a probe^32^. Etiolated seedlings were pre-treated with each of the inhibitors at 10 μM for 30 min and then illuminated with or without blue light at 100 μmol m^−2^ s^−1^ for 1 min.

To identify the protein kinase(s) that regulate blue light-dependent phosphorylation of a penultimate Thr of the H^+^-ATPase in guard cells, we first screened for kinase inhibitors that suppress the phosphorylation from the SCREEN-WELL kinase inhibitor library (80 inhibitors) (Enzo Life Sciences). Epidermal fragments from wild-type (WT) leaves were illuminated by red light (50 μmol m^−2^ s^−1^) with or without blue light (10 μmol m^−2^ s^−1^), after which phosphorylation of the guard cell H^+^-ATPase was detected by an immunohistochemical method using specific antibodies against phosphorylated Thr of the H^+^-ATPase^23^. Blue light stimulated more phosphorylation compared to red light (Fig. 1a,b). We found that the phosphorylation was effectively suppressed by treatment with four inhibitors: Tyrphostin 9, Sphingosine, GW5074, and BML-265. We next investigated the effects of the inhibitors on blue light-dependent stomatal opening in leaf epidermis (Fig. 1c). Stomata closed in the dark and opened in response to light (red light, 50 μmol m^−2^ s^−1^ and blue light, 10 μmol m^−2^ s^−1^) under treatment with DMSO. In contrast, tyrphostin 9, sphingosine, and GW5074 effectively suppressed light-dependent stomatal opening. BML-265 also suppressed light-dependent stomatal opening, but not significantly.

Next, we investigated whether or not the four inhibitors affect phototropin kinase activity. Blue light induced the autophosphorylation of phot1, which was detected by a 14-3-3 protein binding in protein blotting (Fig. 1d: DMSO). Staurosporine, a general protein kinase inhibitor effectively inhibited the autophosphorylation of phot1, as reported previously^22^. In contrast, the four inhibitors had no effect on autophosphorylation (Fig. 1d). The results suggested that targets of the kinase inhibitors may regulate blue light-dependent stomatal opening without affecting phototropin activity.

### Presumption of the potential inhibitor targets in Arabidopsis

**Figure 2:**
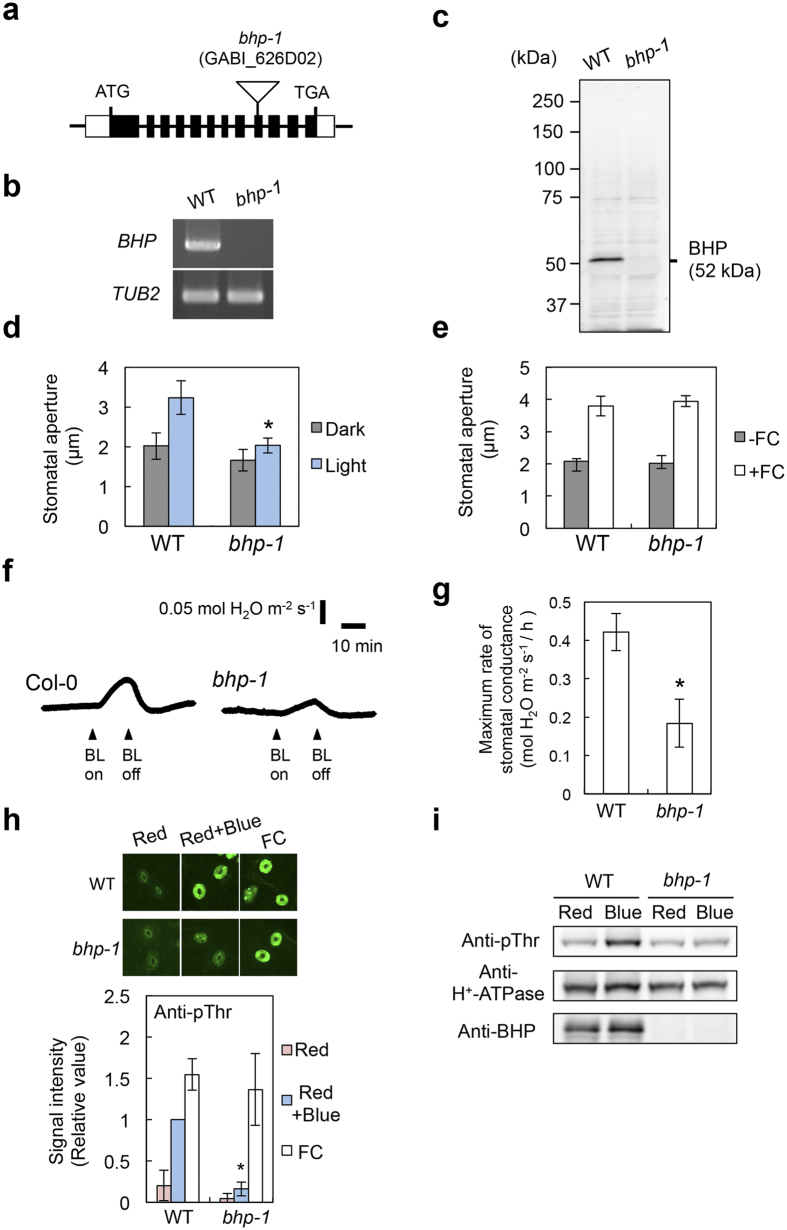
*bhp-1* exhibits impairment in stomatal responses in response to blue light. (**a**) Schematic representation of the *BHP* gene (*At4g18950*) and position of the T-DNA insertion in the *bhp-1* mutant. Boxes and lines indicate exons and introns, respectively. (**b**,**c**) Expressions of the *BHP* mRNA and BHP protein in wild-type (WT) and *bhp-1* were determined by RT-PCR (**b**) and immunoblotting (**c**), respectively. *TUBULIN2 (TUB2*) was used as an internal control in (**b**). (**d**,**e**) Stomatal opening in response to blue light (**d**) or fusicoccin (FC) (**e**) in WT and *bhp-1* leaves. FC was applied to the epidermis (10 μM) in the dark for 3 h. Values represent means ± SD (*n* = 3); measurement of 30 stomata in each experiment. *indicates values that statistically differ from the corresponding WT (Student’s *t* test; *p < 0.05). (**f**) The change in stomatal conductance in response to blue light in intact leaves from WT and *bhp-1*. Leaves of dark-adapted plants were irradiated by red light (600 μmol m^−2^ s^−1^), then superimposed by blue light (5 μmol m^−2^ s^−1^) for 15 min. Arrowheads indicate the start and end of blue light irradiation. (**g**) Maximum rate of stomatal conductance in response to blue light in WT and *bhp-1* leaves. Data represent means ± SD (*n = *4). *indicates values that statistically differ from WT (Student’s *t* test; *p < 0.01). (**h**) Immunohistochemical detection of H^+^-ATPase phosphorylation in response to blue light and 10 μM FC in WT and *bhp-1* guard cells. Values indicate means ± SD (*n = *4), with measurement of 30 stomata in each experiment. *indicates values that statistically differ from the corresponding WT (Student’s *t* test; *p < 0.01). (**i**) Blue light-dependent H^+^-ATPase phosphorylation in WT and *bhp-1* guard cell protoplasts (GCPs). GCPs were illuminated by red light (Red: 600 μmol m^−2^ s^−1^, 30 min), then blue light (Blue: 100 μmol m^−2^ s^−1^, 30 sec) was superimposed on the red light. Immunoblots of the phosphorylated H^+^-ATPase, total H^+^-ATPase, and BHP were performed using individual antibodies.

In mammalian cells, Tyrphostin 9, Sphingosine, GW5074, and BML-265 inhibited platelet-derived growth factor receptor kinase (PDGFRK), protein kinase C (PKC), c-Raf, and epidermal growth factor receptor kinase (EGFRK), respectively. We predicted the inhibitor targets in Arabidopsis by BLAST search based on amino acid sequences of the mammalian kinases. We found that all of the Arabidopsis kinases similar to PDGFRK, c-Raf, and EGFRK belong to a Raf-like kinase subfamily of the MAPKKK family (Supplementary Table 1). In Arabidopsis, the kinase subfamily contains 48 members^24^,^25^ (Supplementary Fig. 1a). However, the physiological functions of the members are largely unknown^26^. We suspected that some members in the subfamily mediate blue light-dependent stomatal opening. To investigate this, we first focused on the expression levels of the subfamily genes in guard cells because most signaling components for stomatal movements, such as ABA INSENSITIVE 1 (ABI1), ABI2, OPEN STOMATA 1 (OST1), OPEN STOMATA2/ARABIDOPSIS H^+^-ATPase 1 (OST2/AHA1), PHOT1, HIGH LEAF TEMPERATURE 1 (HT1), SLOW ANION CHANNEL-ASSOCIATED 1 (SLAC1), and BLUS1 are highly expressed in guard cells^27^,^28^,^29^. Thus, we searched for guard cell-rich Raf-like kinases in a public microarray database eFP browser (http://bar.utoronto.ca/efp/cgi-bin/efpWeb.cgi?dataSource=Guard_Cell). Among 48 genes of the members, 12 were highly expressed in guard cells (Supplementary Fig. 1b). It had been demonstrated that one of the 12 members, HT1, regulates stomatal movements in response to CO_2_ and the *ht1* mutant exhibits normal stomatal opening in response to blue light^30^,^31^. Therefore, we obtained T-DNA inserted knockout mutants of the other 11 members, excluding HT1 (Supplementary Fig. 2 and Fig. 2b).

### Identification of BHP that regulates blue light-dependent stomatal opening

**Figure 3:**
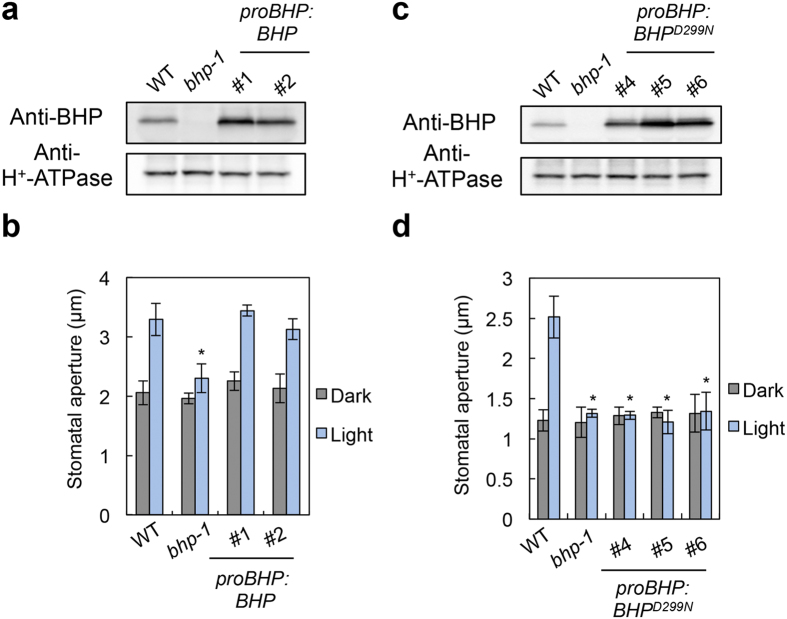
BHP kinase activity is required for stomatal opening in response to blue light. Functional complementation of stomatal opening by the introduction of the WT *BHP* gene (**a**,**b**) or inactive *BHP*^*D299N*^ gene (**c**,**d**) into the *bhp-1* mutant. ^#^ represents an independent transgenic line. (**a**,**c**) Expression of BHP protein in leaves of transgenic plants used for complementation experiments in (**b**,**d**). Immunoblots of BHP and H^+^-ATPase were performed using proteins prepared from rosette leaves of 3-week-old plants. H^+^-ATPase was used as a loading control. (**b**,**d**) Stomatal aperture in the transgenic lines. Data represent means ± SD (*n* = 4); measurement of 30 stomata in each experiment. *indicates values that statistically differ from the corresponding WT (Student’s *t* test; *p < 0.01).

We determined the blue light-dependent stomatal opening in the knockout mutants (Supplementary Fig. 3). Although most of the mutants showed stomatal opening similar to WT, two knockout mutants of the C1 group members, GABI_626D02 and SALK_002267, lost stomatal opening in response to blue light (Fig. 2d and Supplementary Fig. 3). These results indicate that two genes of the Raf-like kinases, *At4g18950* and *At1g14000*, are required for blue light-dependent stomatal opening. We named *At4g18950* as BLUE LIGHT-DEPENDENT H^+^-ATPASE PHOSPHORYLATION (BHP). T-DNA of the GABI_626D02 (*bhp-1* mutant) was inserted into the ninth exon of the *BHP* gene (Fig. 2a). *BHP* mRNAs and BHP proteins were not detected in the *bhp-1* mutant (Fig. 2b,c), indicating that the *bhp-1* is a null mutant. Blue light-dependent increase in stomatal conductance was also reduced by about 60% in the *bhp-1* mutant (Fig. 2f,g). We next determined the blue light-dependent phosphorylation of the guard cell H^+^-ATPase using the immunohistochemical method in WT and *bhp-1* epidermis (Fig. 2h). As expected, the phosphorylation of H^+^-ATPase in guard cells was lost in the *bhp-1* mutant. This result was similarly obtained by immunoblotting using guard cell protoplasts (GCPs) (Fig. 2i). The amount of H^+^-ATPase in *bhp-1* guard cells was similar to that in WT. Consistent with the results, another allelic knockout mutant, *bhp-2*, also lost stomatal opening in response to blue light (Supplementary Fig. 4). The impairment of the stomatal opening in the *bhp-1* mutant was completely recovered by introduction of the WT *BHP* gene (Fig. 3a,b). These results demonstrate that the stomatal phenotype is derived from the *BHP* gene knockout.

Next, we investigated whether or not stomatal opening requires the protein-kinase activity of BHP. We expressed an inactive kinase of BHP^D299N^ 32,33 under the control of the *BHP* promoter in the *bhp-1* mutant (Fig. 3c,d). The mutant BHP^D299N^ protein in transgenic plants did not restore stomatal opening in response to blue light (Fig. 3d). These results suggest that BHP functions as a protein kinase upstream of H^+^-ATPase phosphorylation and positively regulates blue light-dependent stomatal opening. Therefore, we predicted that the selected inhibitors suppress H^+^-ATPase phosphorylation through the inhibition of BHP. To examine the effects of the inhibitors on BHP kinase activity, we attempted to measure activity using recombinant GST-BHP and BHP-GST produced by both *E. coli* and *in vitro* translation systems. However, we could not detect BHP activity in the systems or estimate the effects of inhibitors on kinase activity.

The H^+^-ATPase activator fusicoccin (FC) irreversibly induces phosphorylation of the penultimate Thr of H^+^-ATPase and stomatal opening, even in the dark^34^. Stomata in both WT and *bhp-1* mutant showed large opening in response to FC (Fig. 2e). Furthermore, FC similarly induced phosphorylation of the guard cell H^+^-ATPase in both *bhp-1* mutant and WT (Fig. 2h). These results indicated that BHP regulates phosphorylation of a penultimate Thr of the H^+^-ATPase in response to blue light, but is not involved in FC-induced phosphorylation.

In the knockout mutant of *At1g14000*, SALK_002267, the phosphorylation of the guard cell H^+^-ATPase was normally observed in response to blue light and FC (Supplementary Fig. 5a) and the amount of H^+^-ATPase was not significantly different between WT and mutant guard cells (Supplementary Fig. 5b). Stomata openings in SALK_002267 were smaller than those in WT in response to FC (Supplementary Fig. 5c). The results indicate that a Raf-like kinase At1g14000 is involved in stomatal opening, but is not required for H^+^-ATPase phosphorylation.

### Characterization of BHP

**Figure 4:**
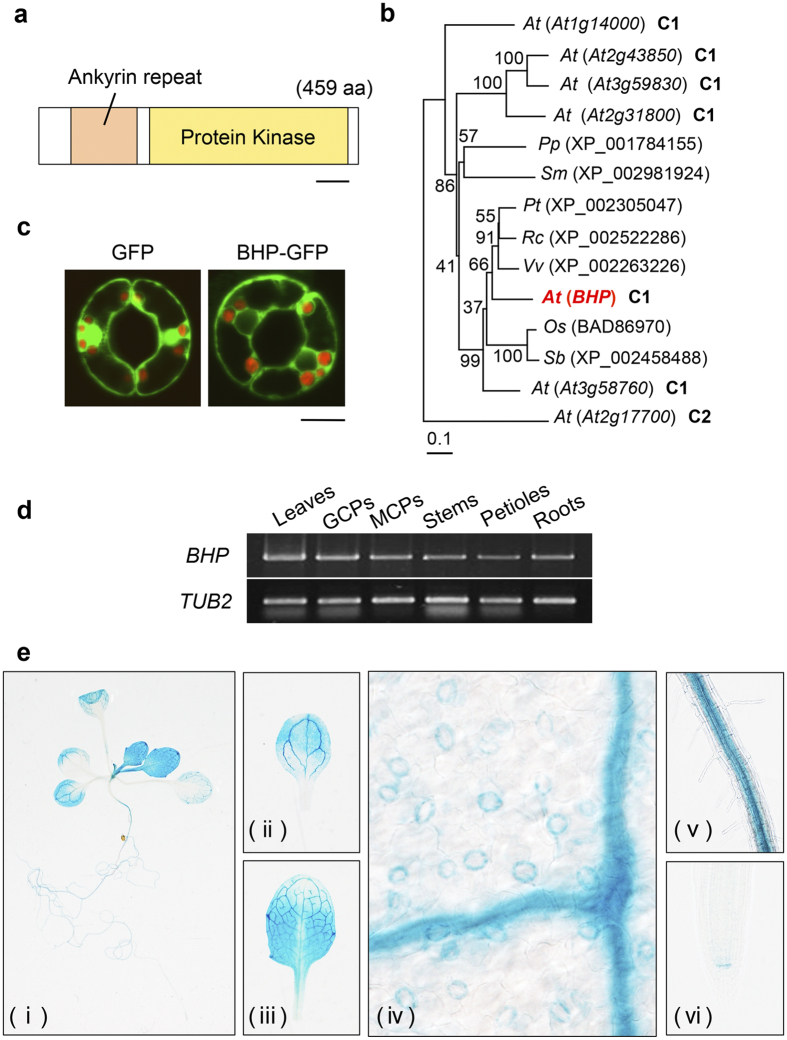
Characteristics of BHP. (**a**) Schematic representation of the BHP protein. BHP protein has an ankyrin repeat and a protein kinase domain. Scale bar represents 50 aa. (**b**) Phylogenetic relationships between BHP and homologous proteins from *Arabidopsis thaliana* and other plant species. The alignment was performed using the ClustalW program with amino acid sequences from the kinase domain from *Arabidopsis thaliana (At*), *Oryza sativa (Os*), *Populus trichocarpa (Pt*), *Physcomitrella patens (Pp*), *Ricinus communis (Rc*), *Sorghum bicolor (Sb*), *Selaginella moellendorffii (Sm*), and *Vitis vinifera (Vv*). The tree was created using MEGA6 software with the neighbor-joining method. The Arabidopsis BHP is shown in bold red letters. The numbers next to the branches are bootstrap values (1,000 replicates). The scale bar represents 0.1 substitutions per site. (**c**) Subcellular localization of GFP or BHP-GFP protein in guard cells. Fluorescent images from GFP (green) were obtained using a confocal laser microscope from transgenic plants expressing GFP or BHP-GFP under the control of the 35 S promoter. Chlorophyll autofluorescence (red) was obtained and shown. Scale bar represents 10 µm. (**d**) Expression of *BHP* determined by RT-PCR analysis. RNA was isolated from leaves, guard cell protoplasts (GCPs), mesophyll cell protoplasts (MCPs), stems, petioles, and roots of 5-week-old WT plants. *TUB2* was used as an internal control. (**e**) Expression pattern of *BHP* determined using the promoter-GUS assay. The *GUS* reporter gene was expressed in transgenic plants under the control of the *BHP* promoter. (i) Whole plant, (ii) cotyledon, (iii) true leaf, (iv) close-up image of true leaf, (v) root, and (vi) primary root tip.

BHP is a member of the C1 group of the Raf-like kinase subfamily, and the functions of BHP remain unclear. BHP has an ankyrin repeat domain that acts in protein-protein interactions in the N-terminus and a protein kinase domain in the C-terminus (Fig. 4a). We found that the BHP homologous proteins are widely conserved in land plants such as *Populus trichocarpa, Ricinus communis, Vitis vinifera, Oryza sativa, Sorghum bicolor, Physcomitrella patens*, and *Selaginella moellendorffii* (Fig. 4b), and may have an identical function in stomatal opening.

We determined the subcellular localization of the BHP protein using transgenic plants expressing the *BHP-GFP* gene under control of the 35S promoter. The fluorescence signal from BHP-GFP was observed in the cytosol of guard cells (Fig. 4c). We note that the signal from GFP alone was observed in both the cytosol and nucleus. RT-PCR analysis was performed using total RNA from rosette leaves (leaves), GCPs, mesophyll cell protoplasts (MCPs), inflorescence stems (stems), petioles, and roots (Fig. 4d). The *BHP* mRNAs were ubiquitously detected in all these tissues, including guard cells. We further determined the *BHP* expression using transgenic plants expressing the *GUS* reporter gene under control of the native *BHP* promoter (Fig. 4e). In accord with the results of RT-PCR, GUS staining was observed in guard and mesophyll cells, leaves, petioles, and roots. Especially, the stain was strongly observed in vascular tissues and guard cells (Fig. 4e; ii, iii, iv, v). Together, these results suggested that BHP functions not in the nucleus, but in the cytosol of guard cells, to induce stomatal opening.

To investigate whether BHP functions generally in phots-mediated blue light responses, we examined other blue light responses, such as phototropism, chloroplast movement, and leaf flattening, in the *bhp-1* mutant (Supplementary Fig. 6)^35^. The *bhp-1* mutant exhibited similar responses as in WT plants. These results indicate that BHP specifically mediates stomatal opening among phots-mediated blue light responses despite its ubiquitous expression.

### Functional location of BHP in signaling for stomatal opening

**Figure 5:**
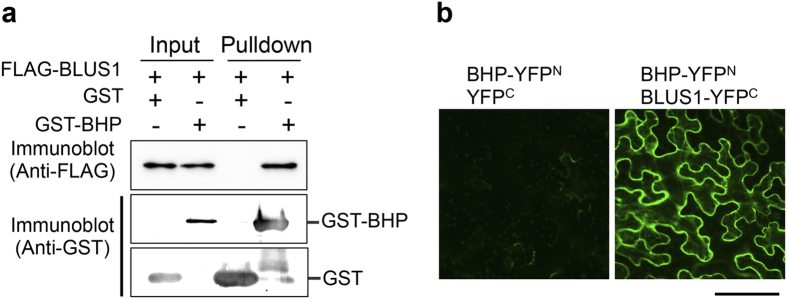
BHP interacts with BLUS1 both *in vitro* and *in vivo*. (**a**) *In vitro* pull-down assay of the interaction of BHP with BLUS1. Both proteins were expressed in *E. coli*. Extract from the *E. coli* cells expressing GST or GST-BHP was mixed with that expressing FLAG-BLUS1 and reacted with glutathione-Sepharose 4B beads. The proteins on the beads were subjected to SDS-PAGE and then immunoblotted using anti-GST and anti-FLAG antibodies. (**b**) *In vivo* interaction of BHP with BLUS1 determined by a bimolecular fluorescence complementation (BiFC) assay. BLUS1 constructs were co-transformed with BHP into *Nicotiana benthamiana* leaves. The reconstituted fluorescent signal was observed using a confocal laser microscope. YFP^N^ and YFP^C^ represent the N- and C-terminal halves of the YFP protein, respectively. Scale bar represents 100 µm.

To investigate the position of BHP in guard cell signaling, we examined the protein-protein interactions between BHP and the known signaling components for stomatal opening using an *in vitro* pull-down assay. Recombinant GST-BHP was used as bait and incubated with recombinant FLAG-BLUS1, FLAG-PP1, or microsomal proteins from the Arabidopsis etiolated seedlings. BHP did not interact with phot1 and the H^+^-ATPase from microsomal membranes, but interacted with purified BLUS1 and PP1 *in vitro* (Fig. 5a and Supplementary Fig. 7). We further confirmed the *in vivo* interactions among the proteins by bimolecular fluorescence complementation (BiFC) assay in *Nicotiana benthamiana* leaves (Fig. 5b and Supplementary Fig. 8a). In agreement with the results of pull-down assays, BHP interacted with BLUS1 in plant cells. Furthermore, we found that BHP interacts with phot1 and phot2. In contrast, BHP does not interact with the H^+^-ATPase isoforms, AHA1 and AHA2, although AHA1 and AHA2 show a positive interaction with each other in the present experimental conditions (Supplementary Fig. 8b).

BLUS1 is a substrate for phot kinases in guard cells^18^. Therefore, BHP may be a substrate of BLUS1 kinase. To test this possibility, we attempted to detect the phosphorylation of BHP by BLUS1 *in vitro* using recombinant proteins produced by both *E. coli* and *in vitro* translation systems. However, we could not detect BLUS1 activity in the systems nor evaluate the target relationship between BLUS1 and BHP.

## Discussion

To identify a novel protein kinase involved in blue light-dependent stomatal opening, we first screened kinase inhibitors that suppress both the blue light-dependent phosphorylation of guard cell H^+^-ATPase and stomatal opening. We searched the potential Arabidopsis targets of the inhibitors based on amino acid sequence homology with mammalian targets and identified BHP. Based on phenotypic analyses of the *bhp* mutants and transgenic *bhp-1* plants expressing WT and inactive BHP, we concluded that BHP functions as a novel positive regulator of blue light-dependent stomatal opening and BHP kinase activity is required for this response.

In the *bhp-1* mutant, blue light-dependent phosphorylation of the H^+^-ATPase was strongly impaired, but FC-induced phosphorylation was similar as in WT (Fig. 2h). Identical protein kinase is most likely to phosphorylate the H^+^-ATPase in response to both blue light and FC^34^. These results suggest that BHP does not directly phosphorylate the H^+^-ATPase. In agreement with this, BHP did not interact with the H^+^-ATPase both *in vitro* and *in vivo* (Supplementary Fig. 7b and 8). There may be an unknown protein kinase that directly phosphorylates the H^+^-ATPase in stomatal opening.

The interaction of BHP with phots was observed *in vivo* but not *in vitro* (Supplementary Figs 7a and 8). Thus, BHP and phots may be in the same complex and interact via other scaffold proteins in plant cells. Interaction of BHP with PP1 could not be evaluated by our BiFC assay, because interaction of PP1 with a regulatory subunit, INHIBITOR3, was not observed in this experiment (see Methods). Because BHP interacted with PP1 *in vitro* (Supplementary Fig. 7c), BHP may also interact with PP1 in guard cells. Clarification of the relationship between BHP and the known components, and identification of the unknown binding partner of BHP, will clarify the actual role of BHP in blue light signaling in guard cells.

The promoter GUS and RT-PCR analyses indicated that *BHP* expression is ubiquitous (Fig. 4d,e). The present results indicate that BHP specifically mediates stomatal opening among the phots-mediated blue light responses (Supplementary Fig. 6). The identity of guard cell-signaling may be determined by BLUS1, which is expressed only in guard cells^18^. BHP may be involved in other physiological responses in tissues other than guard cells. Further investigations are needed to clarify the physiological roles of BHP in other tissues.

BHP belongs to the Raf-like kinase subfamily in the MAPKKK family, which is different from the typical MAPKKKs involved in the MAPK cascade. However, functions of the Raf-like kinases remain unclear^36^,^37^. MAPKKK family is divided into three clades (A to C), and members of clade B and C belong to the Raf-like kinases in Arabidopsis^25^. The C clade of Raf-like kinases (C1 to C7 groups) is the largest clade in the MAPKKK family, and BHP and At1g14000 belong to the C1 group. In the present study, we showed that both proteins mediate blue light-dependent stomatal opening. Moreover, HT1 (C5 group) acts as a negative regulator of high CO_2_-induced stomatal closure^30^. These results suggested that C clade members of Raf-like kinases control stomatal aperture in response to light and CO_2_ to adapt plant transpiration rates to the environment.

BHP and At1g14000 function differently in blue light-dependent stomatal opening; At1g14000 did not mediate blue light-dependent phosphorylation of the H^+^-ATPase without affecting the H^+^-ATPase amount (Supplementary Fig. 5a,b). Stomatal opening by FC was reduced in SALK_002267 compared with WT (Supplementary Fig. 5c), suggesting that signaling for stomatal opening is decreased downstream of H^+^-ATPase activation in the mutant guard cells. Accordingly, previous studies indicated that At1g14000 (VH1-INTERACTING KINASE; VIK) is involved in auxin and brassinosteroid signaling, and functions in glucose import into the vacuole at the tonoplast^38^,^39^. Ion transports into the vacuole have crucial roles in increasing guard-cell volume for stomatal opening^40^. These results suggested that BHP functions in the guard cell cytosol to regulate H^+^-ATPase activity, and VIK may function in the guard cell tonoplast to regulate stomatal opening. There may be unknown C-clade members of Raf-like kinases that function in guard cells. However, further analyses are required to understand the Raf-like kinase functions and signaling for stomatal movement.

## Methods

### Plant materials and growth conditions

Plants of *Arabidopsis thaliana*, ecotype Columbia-0 (Col), were used as WT. All T-DNA insertion mutants (Col background) were obtained from Arabidopsis Biological Resource Center and Nottingham Arabidopsis Stock Center. Arabidopsis and *Nicotiana benthamiana* plants were grown on soil for 3 to 5 weeks at 24 °C under relative humidity of 55–70% in the growth room. The growth room was set under a 16 h white light (50 μmol m^−2^ s^−1^)/8 h dark cycle.

### Immunohistochemical staining of the guard cell H^+^-ATPase

Visualization of the phosphorylation and the amount of guard cell H^+^-ATPase was performed and the fluorescent signal intensities were quantified using ImageJ software (http://imagej.nih.gov/ij/) according to our previous method^23^.

### Stomatal aperture and conductance

Stomatal aperture was measured using epidermis from the rosette leaves according to previous methods^23^,^32^. Epidermal fragments were incubated in reaction buffer 1 containing 5 mM MES/bistrispropane (pH 6.5), 50 mM KCl, and 0.1 mM CaCl_2_ and illuminated with light (red light at 50 μmol m^−2^ s^−1^ and blue light at 10 μmol m^−2^ s^−1^) or treated with FC (10 μM) in the dark for 3 h (Figs 1c,2d,e and 3b,d; Supplementary Figs 3 and 4c). The protein kinase inhibitors (10 μM) were pretreated with the epidermis 30 min before light illumination. To determine stomatal opening by FC in SALK_002267, the epidermis was incubated in reaction buffer 2 containing 5 mM MES/bistrispropane (pH 6.5), 10 mM KCl, and 0.1 mM CaCl_2_ with or without FC at the indicated concentrations in the dark for 3 h (Supplementary Fig. 5c).

Stomatal conductance in intact leaves of WT and *bhp-1* was determined according to previous methods^5^,^32^.

### Isolation of guard and mesophyll cell protoplasts

Guard and mesophyll cell protoplasts (GCPs and MCPs) were isolated from rosette leaves of Arabidopsis plants, as described previously^6^ with minor modifications. We used Macerozyme R-10 (Yakult) instead of pectolyase Y-23 in the enzyme mixture. The obtained protoplasts were suspended in 400 mM mannitol and 1 mM CaCl_2_, and were kept on ice in the dark until use.

### Preparation of microsomes

The microsomes from etiolated seedlings of *A. thaliana* were prepared using a pervious method^22^ with minor modifications. The 3-day-old etiolated seedlings were homogenized with extraction buffer (50 mM MOPS-KOH pH7.5, 100 mM NaCl, 2.5 mM EDTA, 1 mM PMSF, 20 μM Leupeptin, 1 mM DTT). The homogenate was centrifuged at 19,000 × *g* for 8 min at 4 °C. The supernatant was further centrifuged at 100,000 × *g* for 60 min at 4 °C. The obtained microsomal membranes were suspended in extraction buffer and kept on ice until use.

### Detection of phot1 autophosphorylation in etiolated seedlings

Three-day-old etiolated seedlings were grown as described in a previous report^32^. Fifty etiolated seedlings were incubated in reaction buffer 1 with or without each kinase inhibitor (10 μM) in the dark for 30 min. The seedlings were kept in the dark or irradiated with blue light (100 μmol m^−2^ s^−1^) for 1 min and then homogenized in ice-cold extraction buffer using a mortar and pestle. The obtained homogenate was centrifuged at 19,000 × *g* for 2 min at 4 °C. The resultant supernatant was solubilized by adding a half volume of SDS solubilization buffer containing 4.5% SDS, 30% sucrose, 22.5% β-mercaptoethanol, 0.018% Coomassie Brilliant Blue, 4.5 mM EDTA, and 45 mM Tris-HCl (pH 8.0). The protein samples were boiled at 95 °C for 5 min and subjected to SDS-PAGE. Immunoblotting of phot1 and protein blotting of phot1 using GST-14-3-3 protein were performed according to previously reported methods^22^,^32^. Experiments repeated three times on separate occasions gave similar results.

### Antibodies

Polyclonal antibodies against the conserved catalytic domain of AHA2 (Anti-H^+^-ATPase), phosphorylated penultimate Thr (Anti-pThr), and the N-terminus of phot1 (Anti-phot1) were used for immunostaining and immunoblotting, as reported previously^5^,^23^,^41^. Anti-FLAG antibody was purchased (Sigma-Aldrich) and used. Anti-BHP or anti-GST antibody was raised against the recombinant BHP or GST protein as an antigen in a rabbit (Medical & Biological Laboratories). Full-length cDNA of *BHP* was amplified by PCR with primers 5′-CGGGATCCATGGAAGAGGATTATCAACAGC-3′ and 5′-CGGGATCCTCACAAATGTGAACCGGATG-3′ and cloned into the *Bam*HI site of the pGEX-2T vector (GE Healthcare). The resulting construct or empty vector was transformed into the *E. coli* BL21 strain. The recombinant BHP protein was expressed as a fusion protein to the glutathione S-transferase (GST-BHP). The fusion protein or GST protein was purified using the glutathione-Sepharose 4B (GE Healthcare). The BHP protein was obtained from the sepharose beads by digestion with thrombin to cut off the GST. GST proteins were eluted from the boundary beads by addition of elution buffer including 50 mM Tris-HCl (pH 8.0) and 10 mM glutathione. The proteins were used for the immunization as antigens.

### Immunoblotting using GCP proteins

Immunoblots to determine phosphorylation and the amount of guard cell H^+^-ATPase were performed according to previous methods^23^ with minor modifications. GCPs were prepared as described above. After light illumination of GCPs, the phosphorylation reaction of H^+^-ATPase was terminated 2.5 min after the start of blue light illumination. The guard cell proteins (50 μg) were solubilized by addition of GCPs-solubilization buffer containing 5 mM MOPS-KOH, 1.75 mM EDTA, 1 mM phenylmethylsulfonyl fluoride (PMSF), 20 μM leupeptin, 1.5% SDS, 15% sucrose, 5% 2-mercaptoethanol, 0.01% Coomassie Brilliant Blue, and 15 mM Tris-HCl (pH 8.0) at room temperature. The protein samples were subjected to SDS-PAGE and then immunoblotted using anti-pThr and anti-H^+^-ATPase. To detect BHP proteins in GCPs, we performed immunoblotting using anti-BHP antibodies as above. For the immunoblotting of BHP, the nitrocellulose membrane was blocked and incubated with the antibody in Blocking One (Nacalai Tesque) instead of a 5% skim milk solution. The anti-BHP antibody was used at a 2,000-fold dilution in the primary antibody. Phosphorylation and the total amount of the H^+^-ATPase were determined by immunoblotting using anti-pThr and H^+^-ATPase antibodies, respectively. Experiments repeated three times on independent occasions gave similar results.

### *In vitro* pull-down assay

The GST-BHP protein was expressed in *E. coli* as described above. FLAG-tagged BLUS1 and -PP1 [TYPE ONE PROTEIN PHOSPHATASE 4; TOPP4 (At2g39840)] were generated as recombinant proteins from *E. coli* (Fig. 5a and Supplementary Fig. 6c). Microsomal proteins from the etiolated Arabidopsis seedlings were used as a source of the proteins (Supplementary Fig. 6a,b). The *BLUS1* and *TOPP4* cDNA fragments were amplified by RT-PCR from the Col cDNAs using primers 5′-CGGAATTCCATGGCTCGGAACAAGCTCGAGTTC-3′ and 5′-CGGAATTCTTAACCCAAAACACTATCTTTATCAG-3′ for *BLUS1* and 5′-CGGAATTCCATGGCGACGACGACGACG-3′ and 5′-CGGAATTCTCAAATCTTTGTGGACATCATGAACTTG-3′ for *TOPP4*. The PCR products were cloned into the *Eco*RI site of the pFLAG-MAC expression vector (Sigma-Aldrich), and plasmids were introduced into the *E. coli* BL21 strain.

*E. coli* was grown in LB broth at 37 °C for 16 h and the culture was diluted three times with new LB broth. The culture was further grown at 20 °C for 2 h in the presence of 0.5 mM isopropyl-β-D-thiogalactopyranoside. *E. coli* cells were collected and suspended in Tris-buffered saline (TBS) containing 5 mM dithiothreitol (DTT), 1 mM PMSF, and 20 μM leupeptin, and were then disrupted by sonication on ice. After centrifugation at 10,000 × *g* for 10 min at 4 °C, each supernatant from *E. coli* lines was mixed and added with 0.5% TritonX-100. The mixtures were reacted with 30 μL of glutathione-Sepharose 4B (GE Healthcare) for 30 min at 4 °C. The beads were washed three times with TBS and then solubilized by the addition of 20 μL of SDS sample buffer [5% (w/v) SDS, 30 mM Tris–HCl pH 8.0, 3 mM EDTA, 30% (w/v) sucrose, 0.012% (w/v) Coomassie Brilliant Blue, 15% (v/v) 2-mercaptoethanol]. The solubilized samples were subjected to SDS-PAGE on a 10% acrylamide gel and immunoblotted using anti-GST or FLAG-antibody (Sigma-Aldrich).

For the pull-down assay using phot1 and the H^+^-ATPase, microsomal membranes were prepared as described above and suspended in TBS containing 5 mM DTT, 1 mM PMSF, 20 μM leupeptin, and 0.5% TritonX-100. The microsomal proteins were reacted with the GST-14-3-3 protein (GF14ϕ) or GST-BHP on glutathione-Sepharose 4B for 2 h at 4 °C. After washing the beads, samples were solubilized in 20 μL of SDS sample buffer and SDS-PAGE was performed on a 9% acrylamide gel. Immunoblotting was performed using anti-GST, -phot1, and -H^+^-ATPase antibodies.

### Expression of *BHP* mRNA by RT-PCR

Total RNAs were extracted from rosette leaves, GCPs, MCPs, stems, petioles, and roots of 5-week-old Col plants using NucleoSpin RNA Plant (Takara). First strand cDNA was synthesized from the RNAs using PrimeScript II 1st strand cDNA Synthesis Kit (Takara). The *BHP* cDNA fragment was amplified from the cDNAs by PCR using primers 5′-ATGGAAGAGGATTATCAACAGC-3′ and 5′-TCACAAATGTGAACCGGATG-3′.*TUB2* fragment was amplified using primers 5′-CATTGTTGATCTCTAAGATCCGTG-3′ and 5′-TACTGCTGAGAACCTCTTGAG-3′, and was used for an internal standard. The primers for amplification of the other genes of Raf-like kinases are listed in Supplementary Table 2.

### Functional complementation experiment

Genomic fragments of the *BHP* gene (5,974 bps), including 5′ and 3′ noncoding regions, were amplified by PCR from genomic DNA of Col using primers 5′-CGGGATCCTGGGTGAAAGTTGACGAACATACTC-3′ and 5′-CGGGATCCCCTCGATCAATAAAGGTCGGCGATC-3′. The DNA fragment was cloned into the *Bam*HI site of the pCAMBIA1300 vector (CAMBIA). To generate a transgenic plant expressing the inactive kinase of the BHP^D299N^ protein, single amino acid substitution was introduced. The aspartic acid is a binding site of Mg^2+^-ATP in protein kinases^33^ and substitution of the aspartic acid with asperagine leads to inactivation of protein kinases^18^,^32^. Nucleotide substitution was introduced into the *BHP* gene in the pCAMBIA1300 vector as templates for inverse PCR. Inverse PCR was performed using the oligonucleotide primers 5′-AACTTTGGAGTAAGCAAGCTTG-3′ and 5′-TGCAACTTTCAGATGCCCTG-3′. The PCR mixtures were treated with *Dpn*I to digest the template DNA and then with T4 polynucleotide kinase to phosphorylate the 5′-ends of the PCR products. The phosphorylated linear DNAs were self-ligated. The vectors were transformed into the *Agrobacterium tumefaciens* GV3101 strain. *Agrobacterium* was transformed into the *bhp-1* mutant by the floral dip method^42^. Transgenic plants were selected by resistance against hygromycin and used for phenotypic analysis of stomatal opening.

### Subcellular localization of BHP

To analyze the subcellular localization of BHP in guard cells, we generated the transgenic plants expressing BHP-GFP under the control of 35S promoter. The full-length cDNA of *BHP* was amplified by PCR using primers 5′-CATGCCATGGAAGAGGATTATCAACAGC-3′ and 5′-CATGCCATGGCCAAATGTGAACCGGATGATG-3′, and was cloned into the *Nco*I site of the CaMV35S-sGFP(S65T)-NOS3′ vector^43^. The cDNA of the *BHP-GFP* was amplified by PCR from the resulting vector using primers 5′-CATATGCCCGTCGACATGGAAGAGGATTATCAACAGC-3′ and 5′-TCAGAATTCGGATCCTTACTTGTACAGCTCGTCCATGCC-3′ and cloned into the pRI 101-AN DNA vector (Takara) using the In-Fusion cloning system (Clontech). The resulting construct was introduced into Arabidopsis plants (Col) using an *Agrobacterium*-mediated transformation method^42^. The transgenic plants were selected based on the kanamycin resistance of the introduced T-DNA. GFP fluorescent signal from guard cells was detected in the epidermis of rosette leaves using a confocal laser microscope (FV10i; Olympus).

### Expression pattern of *BHP* determined by a promoter-GUS assay

The promoter region of *BHP* gene (2,139 bps) was amplified by PCR from the genomic DNA of Col using primers 5′-CAGGCATGCAAGCTTTGGGTGAAAGTTGACGAACATACTC-3′ and5′-AGTCAGATCTACCATTCCAACAAAAACCCTAAAACCCTGAT-3′. The PCR product was cloned into pCAMBIA1303 vector (CAMBIA) using the In-Fusion cloning system. The resulting vector was introduced into Arabidopsis plants (Col) as described above. The transgenic plants were selected by hygromycin resistance and were used for histochemical GUS staining.

The GUS staining was performed as reported previously^44^. The 2- to 3-week-old plants were fixed by 90% acetone for 20 min on ice, and then incubated in the GUS staining buffer {0.5 mg/ ml 5-bromo-5-chloro-3-indolyl-β-d-glucuronide (X-Gluc), 0.5 mM K_5_[Fe(CN)_6_], 0.5 mM K_3_[Fe(CN)_6_], 50 mM sodium phosphate buffer (pH 7.0)} at 37 °C for 16 h. After incubation, the sample was washed with 70% ethanol and fixed with the solution (15% acetic acid and 85% ethanol) until observation. The images of GUS staining were obtained using an upright microscope (Eclipse 50i; Nikon) and a charge-coupled device (CCD) camera (DS-5Mc-L2; Nikon).

### BiFC assay

The cDNAs of *BHP, BLUS1, PHOT1, PHOT2, AHA1, AHA2, TOPP4*, and *INHIBITOR3 (INH3*) were amplified by PCR using the primers listed in Supplementary Table 2. Each amplified cDNA was cloned into the *Sma*I site of both binary vectors pSPYNE173 and pSPYCE(M) using the In-Fusion system. *Agrobacterium* GV3101 strain was transformed with the vector and cultured at 28 °C for 20 h. The agrobacteria were collected and resuspended in infection buffer including 10 mM Mes-KOH (pH 5.6) and 10 mM MgCl_2_. The agrobacteria for BiFC assay and the p19 silencing suppressor strain were mixed and infiltrated into the leaves of *Nicotiana benthamiana* according to previous methods^45^,^46^. YFP fluorescence reconstituted by the BiFC assay was observed 4 days after the infiltration using a confocal laser scanning microscope (FV10i; Olympus). We attempted to detect the interactions between PP1(TOPP4) and BHP or INH3, which binds to PP1, in *in vitro* pull-down, *in vivo* co-immunoprecipitation, and yeast two-hybrid^47^ assays, by co-infiltration with TOPP4-YFP^C^ and BHP-YFP^N^ or INH3-YFP^N^. However, we could not detect significant fluorescent signals even in the co-expression of INH3-YFP^N^/TOPP4-YFP^C^. Thus, we concluded that interactions of PP1 with other proteins could not be observed accurately in our BiFC assay.

### Functional analyses of the phot-mediated responses

Phototropism in etiolated seedlings, chloroplast movement, and leaf flattening were observed according to previous methods^32^.

## Additional Information

**How to cite this article**: Hayashi, M. *et al*. A Raf-like protein kinase BHP mediates blue light-dependent stomatal opening. *Sci. Rep.* **7**, 45586; doi: 10.1038/srep45586 (2017).

**Publisher's note:** Springer Nature remains neutral with regard to jurisdictional claims in published maps and institutional affiliations.

## Supplementary Material

Supplemental Information

## References

[b1] Zeiger E. & Hepler P. K. Light and stomatal function: blue light stimulates swelling of guard cell protoplasts. Science 20, 887–889 (1977). 10.1126/science.196.4292.887 17821809

[b2] Shimazaki K., Doi M., Assmann S. M. & Kinoshita T. Light regulation of stomatal movement. Annu Rev Plant Biol 58, 219–247 (2007). 17209798 10.1146/annurev.arplant.57.032905.105434

[b3] Kinoshita T. & Shimazaki K. Blue light activates the plasma membrane H^+^-ATPase by phosphorylation of the C-terminus in stomatal guard cells. EMBO J 18, 5548–5558 (1999). 10523299 10.1093/emboj/18.20.5548 PMC1171623

[b4] Kinoshita T. . phot1 and phot2 mediate blue light regulation of stomatal opening. Nature 414, 656–660 (2001). 11740564 10.1038/414656a

[b5] Doi M., Shigenaga A., Emi T., Kinoshita T. & Shimazaki K. A transgene encoding a blue-light receptor, phot1, restores blue-light responses in the *Arabidopsis phot1 phot2* double mutant. J Exp Bot 55, 517–523 (2004). 14739272 10.1093/jxb/erh044

[b6] Ueno K., Kinoshita T., Inoue S., Emi T. & Shimazaki K. Biochemical characterization of plasma membrane H^+^-ATPase activation in guard cell protoplasts of *Arabidopsis thaliana* in response to blue light. Plant Cell Physiol 46, 955–63 (2005). 15821287 10.1093/pcp/pci104

[b7] Inoue S., Takemiya A. & Shimazaki K. Phototropin signaling and stomatal opening as a model case. Current Opinion in Plant Biology 13, 587–593 (2010). 20920881 10.1016/j.pbi.2010.09.002

[b8] Kollist H., Nuhkat M. & Roelfsema M. R. Closing gaps: linking elements that control stomatal movement. New Phytol 203, 44–62 (2014). 24800691 10.1111/nph.12832

[b9] Schroeder J. I. . Guard cell signal transduction. Annu Rev Plant Physiol Plant Mol Biol 52, 627–658 (2001). 11337411 10.1146/annurev.arplant.52.1.627

[b10] Palmgren M. G., Larsson C. & Sommarin M. Proteolytic activation of the plant plasma membrane H^+^-ATPase by removal of a terminal segment. J Biol Chem 265, 13423–13426 (1990). 2143184

[b11] Palmgren M. G., Sommarin M., Serrano R. & Larsson C. Identification of an autoinhibitory domain in the C-terminal region of the plant plasma membrane H^+^-ATPase. J Biol Chem 266, 20470–20475 (1991). 1834646

[b12] Regenberg B., Villalba J. M., Lanfermeijer F. C. & Palmgren M. G. C-terminal deletion analysis of plant plasma membrane H^+^-ATPase: yeast as a model system for solute transport across the plant plasma membrane. Plant Cell 7, 1655–1666 (1995). 7580256 10.1105/tpc.7.10.1655 PMC161027

[b13] Palmgren M. G. Plant plasma membrane H^+^-ATPases: Powerhouses for nutrient uptake. Annu Rev Plant Physiol Plant Mol Biol 52, 817–845 (2001). 11337417 10.1146/annurev.arplant.52.1.817

[b14] Fuglsang A. T. . Binding of 14-3-3 protein to the plasma membrane H^+^-ATPase AHA2 involves the three C-terminal residues Tyr^946^-Thr-Val and requires phosphorylation of Thr^947^. J Biol Chem 274, 36774–36780 (1999). 10593986 10.1074/jbc.274.51.36774

[b15] Svennelid F. . Phosphorylation of Thr-948 at the C terminus of the plasma membrane H^+^-ATPase creates a binding site for the regulatory 14-3-3 protein. Plant Cell 11, 2379–2391 (1999). 10590165 10.1105/tpc.11.12.2379 PMC144135

[b16] Spartz A. K. . SAUR Inhibition of PP2C-D phosphatases activates plasma membrane H^+^-ATPases to promote cell expansion in *Arabidopsis*. Plant Cell 26, 2129–2142 (2014). 24858935 10.1105/tpc.114.126037 PMC4079373

[b17] Takemiya A., Kinoshita T., Asanuma M. & Shimazaki K. Protein phosphatase 1 positively regulates stomatal opening in response to blue light in *Vicia faba*. Proc Natl Acad Sci USA 103, 13549–13554 (2006). 16938884 10.1073/pnas.0602503103 PMC1569200

[b18] Takemiya A. . Phosphorylation of BLUS1 kinase by phototropins is a primary step in stomatal opening. Nat Commun 4, 2094; 10.1038/ncomms3094 (2013). 23811955

[b19] Shimazaki K., Kinoshita T. & Nishimura M. Involvement of calmodulin and calmodulin-dependent myosin light chain kinase in blue Light-Dependent H pumping by guard cell protoplasts from *Vicia faba* L. Plant Physiol 99, 1416–1421 (1992). 16669053 10.1104/pp.99.4.1416 PMC1080641

[b20] Shimazaki K., Omasa K., Kinoshita T. & Nishimura M. Properties of the signal transduction pathway in the blue light response of stomatal guard cells of *Vicia faba* and *Commelina benghalensis*. Plant Cell Physiol 34, 1321–1327 (1993).

[b21] Kinoshita T. & Shimazaki K. Evidence for Ca^2+^-dependent protein phosphorylation *in vitro* in guard cells from *Vicia faba* L. Plant Sci 110, 173–180 (1995).

[b22] Kinoshita T. . Blue light- and phosphorylation-dependent binding of a 14-3-3 protein to phototropins in stomatal guard cells of broad bean. Plant Physiol 133, 1453–1463 (2003). 14605223 10.1104/pp.103.029629 PMC300702

[b23] Hayashi M., Inoue S., Takahashi K. & Kinoshita T. Immunohistochemical detection of blue light-induced phosphorylation of the plasma membrane H^+^-ATPase in stomatal guard cells. Plant Cell Physiol 52, 1238–1248 (2011). 21666226 10.1093/pcp/pcr072

[b24] Jonak C., Okrész L., Bögre L. & Hirt H. Complexity, cross talk and integration of plant MAP kinase signalling. Curr Opin Plant Biol 5, 415–424 (2002). 12183180 10.1016/s1369-5266(02)00285-6

[b25] Ichimura K. . Mitogen-activated protein kinase cascades in plants: a new nomenclature. Trends Plant Sci 7, 301–308 (2002). 12119167 10.1016/s1360-1385(02)02302-6

[b26] Shitamichi N., Matsuoka D., Sasayama D., Furuya T. & Nanmori T. Over-expression of MAP3Kδ4, an ABA-inducible Raf-like MAP3K that confers salt tolerance in Arabidopsis. Plant Biotechnol 30, 111–118 (2013).

[b27] Leonhardt N. . Microarray expression analyses of Arabidopsis guard cells and isolation of a recessive abscisic acid hypersensitive protein phosphatase 2C mutant. Plant Cell 16, 596–615 (2004). 14973164 10.1105/tpc.019000 PMC385275

[b28] Vahisalu T. . SLAC1 is required for plant guard cell S-type anion channel function in stomatal signalling. Nature 452, 487–491 (2008). 18305484 10.1038/nature06608 PMC2858982

[b29] Bates G. W. . A comparative study of the *Arabidopsis thaliana* guard-cell transcriptome and its modulation by sucrose. PLoS One 7, e0049641 (2012). 10.1371/journal.pone.0049641 PMC3504121 23185391

[b30] Hashimoto M. . *Arabidopsis* HT1 kinase controls stomatal movements in response to CO_2_. Nat Cell Biol 8, 391–397 (2006). 16518390 10.1038/ncb1387

[b31] Matrosova A. . The HT1 protein kinase is essential for red light-induced stomatal opening and genetically interacts with OST1 in red light and CO_2_ -induced stomatal movement responses. New Phytol 208, 1126–1137 (2015). 26192339 10.1111/nph.13566

[b32] Inoue S. . Blue light-induced autophosphorylation of phototropin is a primary step for signaling. Proc Natl Acad Sci USA 105, 5626–5631 (2008). 18378899 10.1073/pnas.0709189105 PMC2291087

[b33] Hanks S. K. & Hunter T. Protein kinases 6. The eukaryotic protein kinase superfamily: kinase (catalytic) domain structure and classification. FASEB J 9, 576–596 (1995). 7768349

[b34] Kinoshita T. & Shimazaki K. Analysis of the phosphorylation level in guard-cell plasma membrane H^+^-ATPase in response to fusicoccin. Plant Cell Physiol 42, 424–432 (2001). 11333314 10.1093/pcp/pce055

[b35] Christie J. M. Phototropin blue-light receptors. Annu Rev Plant Biol 58, 21–45 (2007). 17067285 10.1146/annurev.arplant.58.032806.103951

[b36] Ning J., Zhang B., Wang N., Zhou Y. & Xiong L. Increased leaf angle1, a Raf-Like MAPKKK that interacts with a nuclear protein family, regulates mechanical tissue formation in the lamina joint of rice. Plant Cell 23, 4334–4347 (2011). 22207574 10.1105/tpc.111.093419 PMC3269869

[b37] Lee S. J., Lee M. H., Kim J. I. & Kim S. Y. Arabidopsis putative MAP kinase kinase kinases Raf10 and Raf11 are positive regulators of seed dormancy and ABA response. Plant Cell Physiol 56, 84–97 (2014). 25324504 10.1093/pcp/pcu148

[b38] Ceserani T., Trofka A., Gandotra N. & Nelson T. VH1/BRL2 receptor-like kinase interacts with vascular-specific adaptor proteins VIT and VIK to influence leaf venation. Plant J 57, 1000–1014 (2009). 19000166 10.1111/j.1365-313X.2008.03742.x PMC2793540

[b39] Wingenter K. . A member of the mitogen-activated protein 3-kinase family is involved in the regulation of plant vacuolar glucose uptake. Plant J 68, 890–900 (2011). 21838775 10.1111/j.1365-313X.2011.04739.x

[b40] Andrés Z. . Control of vacuolar dynamics and regulation of stomatal aperture by tonoplast potassium uptake. Proc Natl Acad Sci USA 111, 1806–1814 (2014). 10.1073/pnas.1320421111 PMC4035970 24733919

[b41] Hayashi Y. . Biochemical characterization of *in vitro* phosphorylation and dephosphorylation of the Plasma Membrane H^+^-ATPase. Plant Cell Physiol 51, 1186–1196 (2010). 20516032 10.1093/pcp/pcq078

[b42] Clough S. J. & Bent A. F. Floral dip: a simplified method for *Agrobacterium*-mediated transformation of *Arabidopsis thaliana*. Plant J 16, 735–743 (1998). 10069079 10.1046/j.1365-313x.1998.00343.x

[b43] Niwa Y., Hirano T., Yoshimoto K., Shimizu M. & Kobayashi H. Non-invasive quantitative detection and applications of non-toxic, S65T-type green fluorescent protein in living plants. Plant J 18, 455–463 (1999). 10406127 10.1046/j.1365-313x.1999.00464.x

[b44] Jefferson R. A., Kavanagh T. A. & Bevan M. W. GUS fusions: beta-glucuronidase as a sensitive and versatile gene fusion marker in higher plants. EMBO J 6, 3901–3907 (1987). 3327686 10.1002/j.1460-2075.1987.tb02730.x PMC553867

[b45] Walter M. . Visualization of protein interactions in living plant cells using bimolecular fluorescence complementation. Plant J 40, 428–438 (2004). 15469500 10.1111/j.1365-313X.2004.02219.x

[b46] Kaiserli E., Sullivan S., Jones M. A. & Feeney K. A. & Christie J. M. Domain swapping to assess the mechanistic basis of *Arabidopsis* phototropin 1 receptor kinase activation and endocytosis by blue light. Plant Cell 21, 3226–3244 (2009). 19880798 10.1105/tpc.109.067876 PMC2782288

[b47] Takemiya A., Ariyoshi C. & Shimazaki K. Identification and functional characterization of inhibitor-3, a regulatory subunit of protein phosphatase 1 in plants. Plant Physiol 150, 144–156 (2009). 19329567 10.1104/pp.109.135335 PMC2675749

