# Supplementary material for: A Raf-like protein kinase BHP mediates blue light-dependent stomatal opening
Source: Sci Rep. 2017 Mar 30;7:45586. doi: 10.1038/srep45586 (PMC5372365; doi:10.1038/srep45586)
Supplement: Supplemental Information [file srep45586-s1.pdf]

Supplementary Information

A Raf-like protein kinase BHP mediates blue light-dependent stomatal opening

Maki Hayashi[1], Shin-ichiro Inoue[1], Yoshihisa Ueno[1,3], Toshinori Kinoshita[1,2,*]

[1]Division of Biological Science, Graduate School of Science, Nagoya University, Chikusa, Nagoya 464-8602, Japan
[2]Institute of Transformative Bio-Molecules (WPI-ITbM), Nagoya University, Chikusa, Nagoya 464-8602, Japan
[3]Current address: Department of Agriculture, Ryukoku University, Seta Oe-cho, Otsu, Shiga 520-2194, Japan

*Corresponding author: Toshinori Kinoshita
Institute of Transformative Bio-Molecules (WPI-ITbM), Nagoya University, Chikusa, Nagoya 464-8602, Japan
Tel/ Fax: +81-52-789-4778
E-mail: kinoshita@bio.nagoya-u.ac.jp

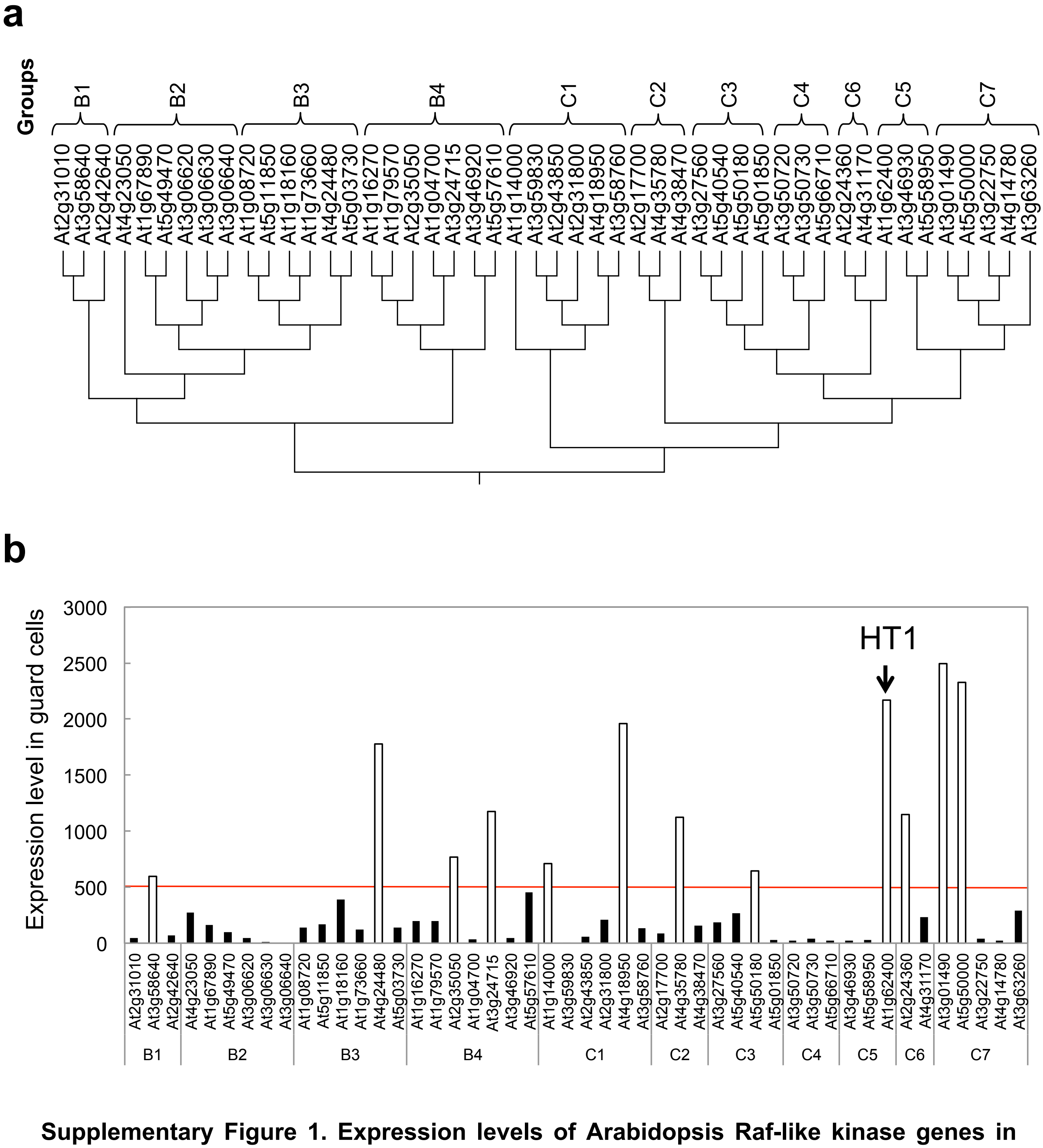

Supplementary Figure 1. Expression levels of Arabidopsis Raf-like kinase genes in guard cells.

(**a**) Phylogenetic relationship in the Arabidopsis Raf-like kinases. The tree was similarly created as described in Fig. 4b. Amino acid sequences of the kinase domains from the members were used. The Raf-like kinases subfamily was classified as B1-B4 and C1-C7 groups, as reported previously[1]. (**b**) Gene expression levels of Raf-like kinases in guard cells. Data of the expression levels in guard cells were obtained from a public microarray database eFP browser (http://bar.utoronto.ca/efp/cgi-bin/efpWeb.cgi?dataSource=Guard_Cell). White columns indicate the high expression genes in guard cells over 500 in the database.

Groups

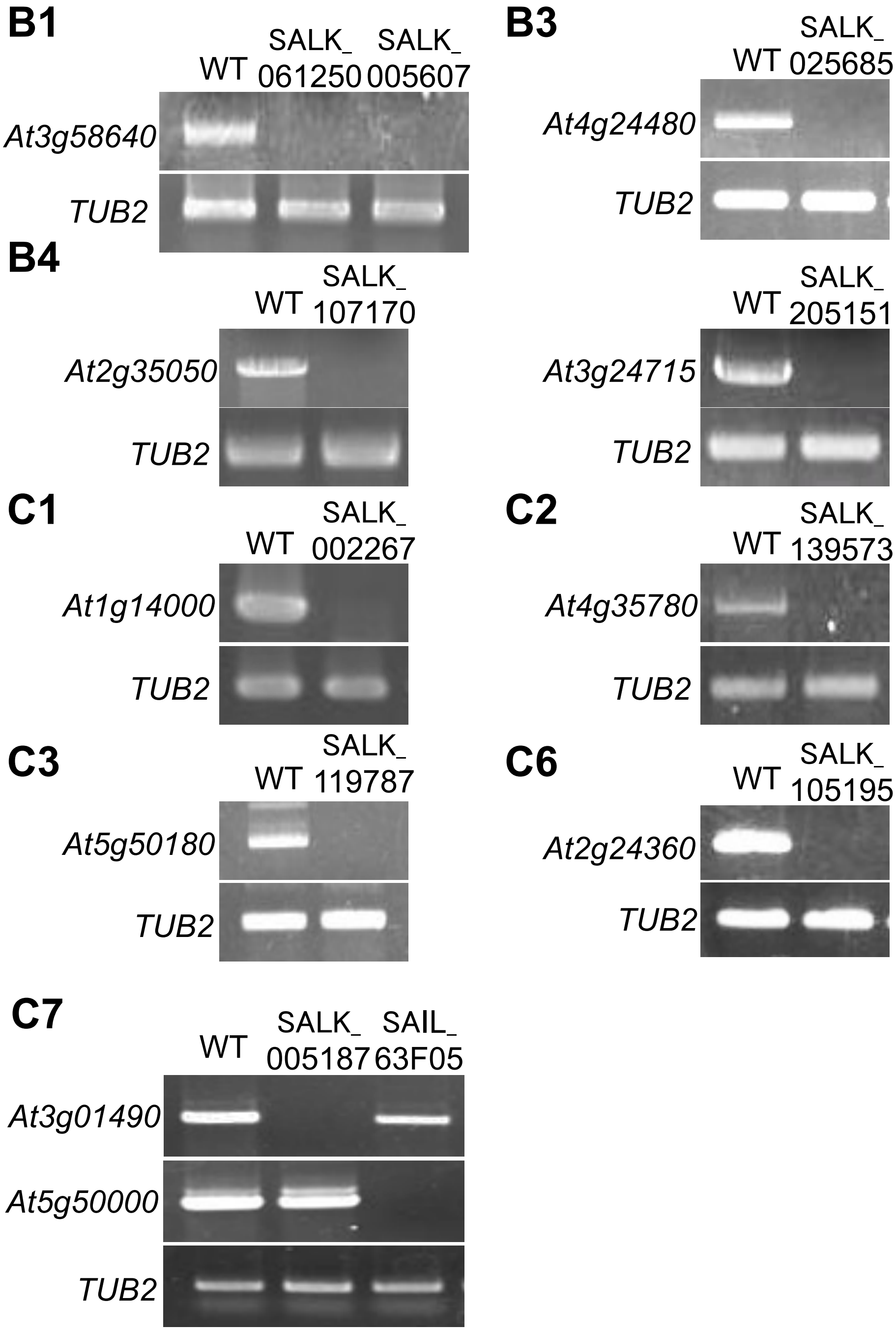

Supplementary Figure 2. Confirmation of gene knockouts in the T-DNA insertion mutants in this study.

RT-PCR was performed using total RNAs from the rosette leaves in wild-type (WT) and T-DNA insertion mutants. All genes were amplified by PCR using the specific primer sets (see Supplementary Table 2). *TUB2* was amplified as an internal control.

Groups

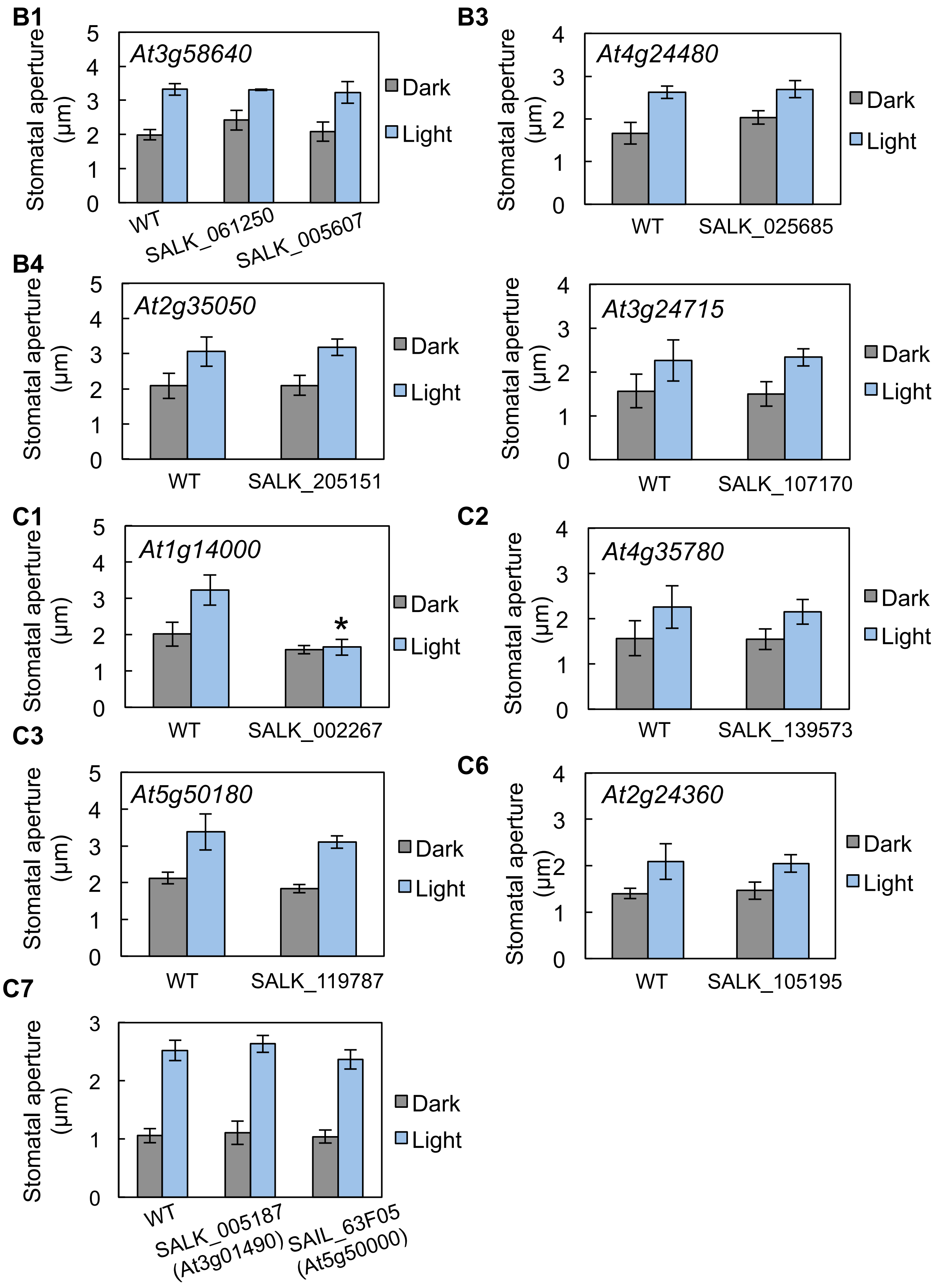

Supplementary Figure 3. Measurements of the blue light-dependent stomatal opening in the T-DNA insertion mutants.

Values represent means±s.d. (n=3); measurement of 30 stomata in each experiment. * indicates values that statistically differ from WT blue light sample (Student's t test; $*p<0.01$).

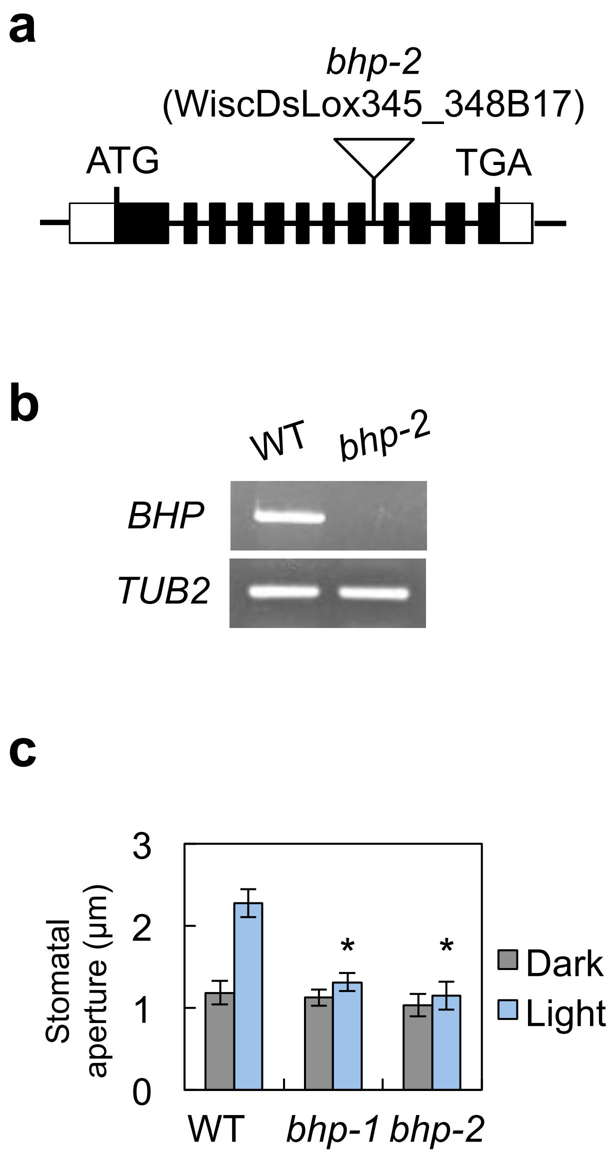

Supplementary Figure 4. Blue light-dependent stomatal opening in T-DNA insertion alleles of *BHP*.
(**a**) Schematic representation of the *BHP* gene (*At4g18950*) and position of the T-DNA insertion in the *bhp-2* mutant. Boxes and lines indicate exons and introns, respectively. (**b**) Knockout of the *BHP* gene in the *bhp-2* mutant. (**c**) Blue light-dependent stomatal opening in leaves from WT, *bhp-1*, and *bhp-2*. The experiment was performed as described in Fig. 2d. Values represent means ±s.d. (n=4); measurement of 30 stomata in each experiment. * indicates values that statistically differ from the corresponding WT (Student's t test; *p<0.01).

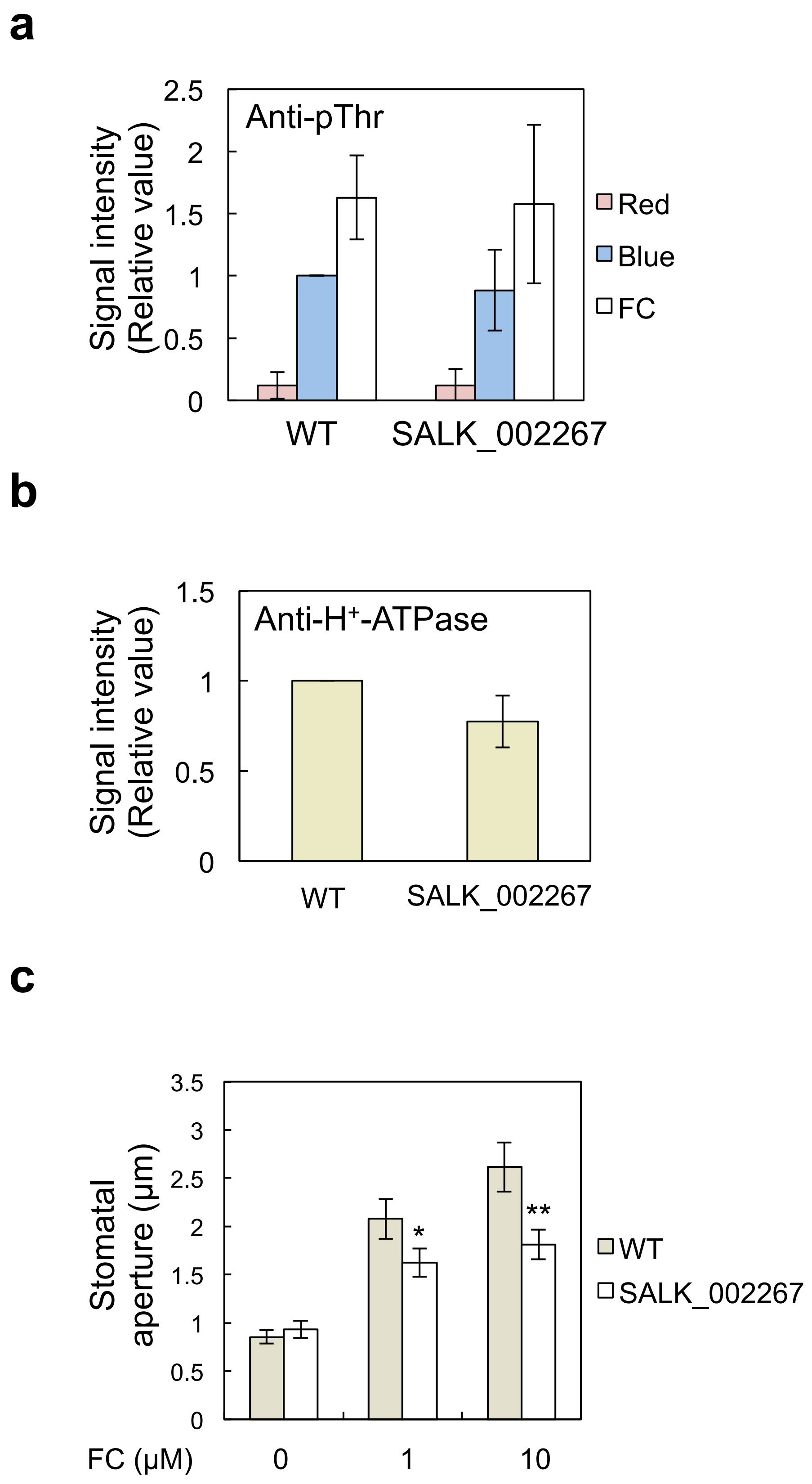
a
Anti-pThr
Signal intensity (Relative value)
2.5
2
1.5
1
0.5
0
Red
Blue
FC
WT
SALK_002267
b
Anti-H+-ATPase
Signal intensity (Relative value)
1.5
1
0.5
0
WT
SALK_002267
c
Stomatal aperture (μm)
3.5
3
2.5
2
1.5
1
0.5
0
*
**
WT
SALK_002267
FC (μM)
0
1
10

Supplementary Figure 5. Immunohistochemical detection of guard cell H^+-ATPase and fusicoccin (FC) induced-stomatal opening in the *At1g14000* knockout mutant.

(**a**) Phosphorylation of the guard cell H^+-ATPase in SALK_002267 in response to blue light and fusicoccin (FC). The immunohistochemical experiments using anti-pThr antibody were performed and are shown in Fig. 2h. Values represent means±s.d. (n=3); measurement of 30 stomata in each experiment. (**b**) Immunohistochemical detection of the amount of guard cell H^+-ATPase in WT and SALK_002267 using anti-H^+-ATPase antibody. The relative signal intensity was expressed as the ratio of the signal intensity from the SALK_002267 mutant to that from WT. Values indicate means ±s.d. (n=3), and measurements of 30 stomata in each experiment. (**c**) Stomatal opening in response to FC in WT and SALK_002267 epidermis. Epidermal fragments from dark-adapted plants were incubated in reaction buffer 2 (see Methods) containing FC at the indicated concentrations for 3 h in the dark. Data represent means±s.d. (n=4). Thirty stomata were measured in each experiment. * and ** indicate values that statistically differ from the corresponding WT (Student's *t* test; $^{**}p<0.01$, $^{*}p<0.05$).

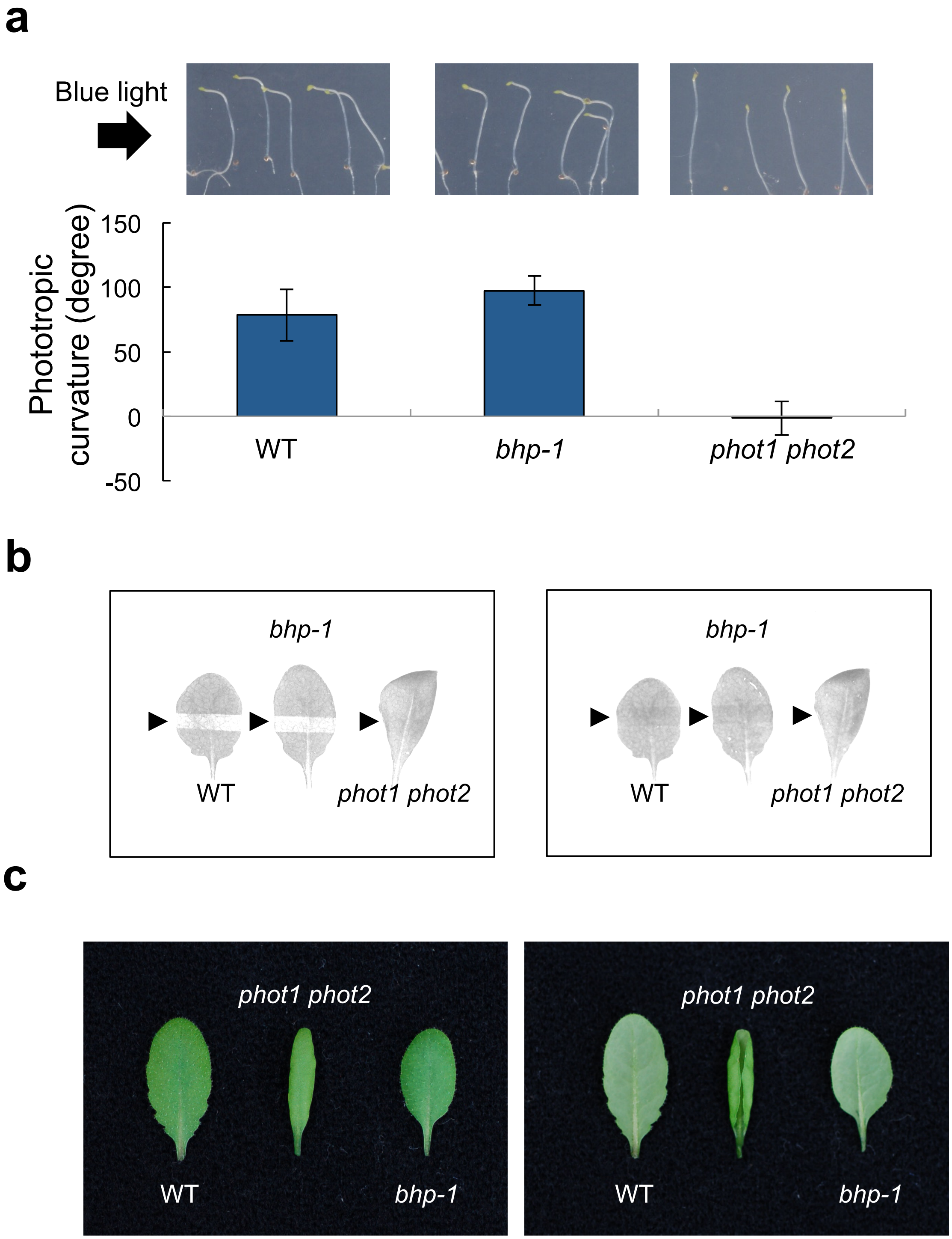

Supplementary Figure 6. Phot-mediated blue light responses in *bhp-1* mutant.
(**a**) Phototropism in WT, *bhp-1*, and *phot1-5 phot2-1* (*phot1 phot2*). Etiolated seedlings were irradiated with unilateral blue light (0.5 μmol m^{-2} s^{-1}) for 14 h. Values represent means±s.d. (*n*=18-19). (**b**) Slit band assays for the chloroplast movement in WT, *bhp-1*, and *phot1 phot2*. Left and right panels indicate the chloroplast avoidance and accumulation responses, respectively. Detached leaves were irradiated with blue light at 100 μmol m^{-2} s^{-1} or 5 μmol m^{-2} s^{-1} for 30 min through a slit to induce the avoidance and accumulation responses, respectively. Arrowheads indicate the irradiated areas. (**c**) Leaf flattening. Rosette leaves were detached from 5-week-old plants, and photos were taken from the adaxial (left panel) and abaxial (right panel) sides.

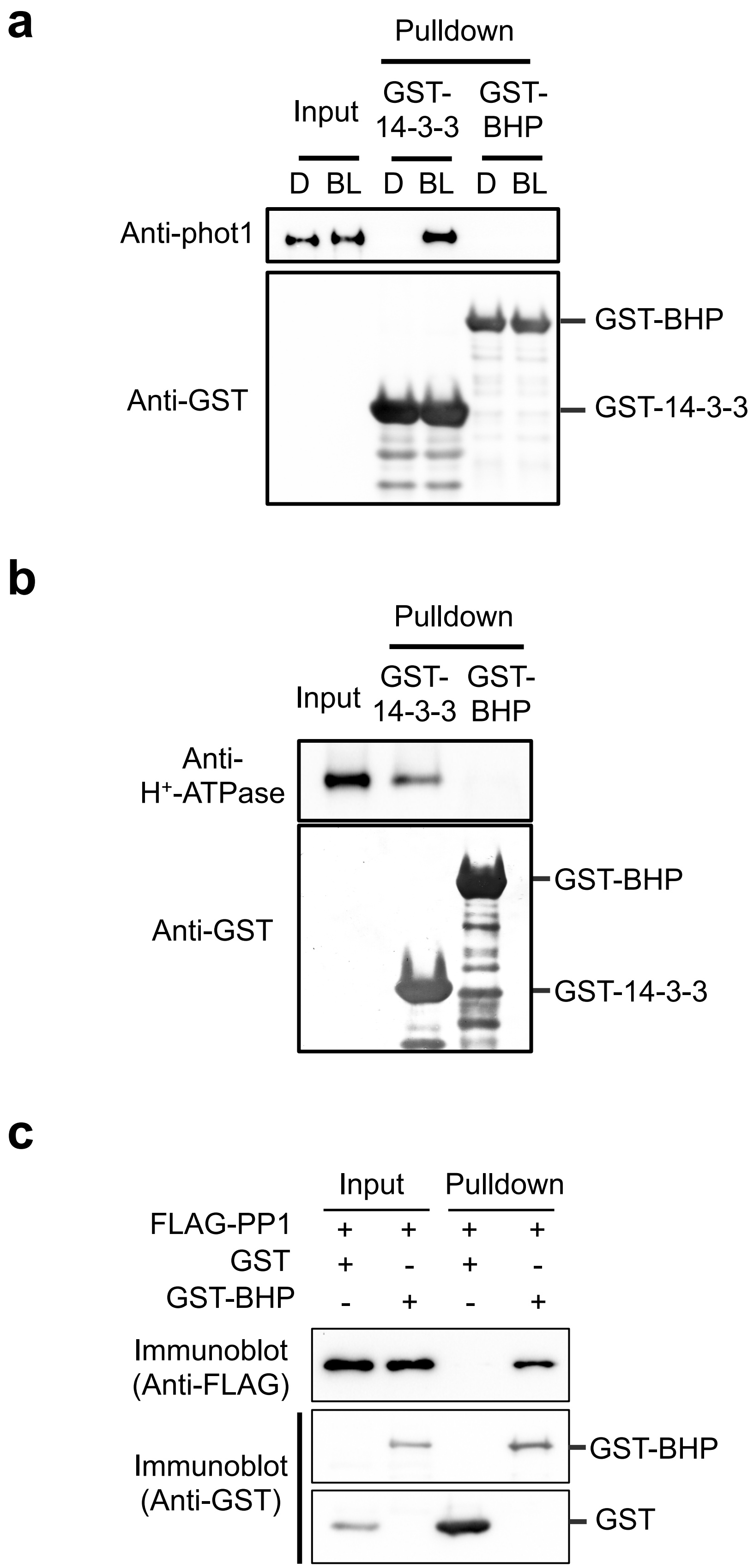
a
Pulldown
Input
GST-14-3-3
GST-BHP
D BL D BL D BL
Anti-phot1
Anti-GST
GST-BHP
GST-14-3-3
b
Pulldown
Input
GST-14-3-3
GST-BHP
Anti-H+-ATPase
Anti-GST
GST-BHP
GST-14-3-3
c
Input
Pulldown
FLAG-PP1 + + + +
GST + - + -
GST-BHP - + - +
Immunoblot (Anti-FLAG)
Immunoblot (Anti-GST)
GST-BHP
GST

Supplementary Figure 7. *In vitro* pull-down assays for interaction of BHP with phot1, H^+-ATPase, and PP1.

(**a,b**) The recombinant GST-14-3-3 protein or GST-BHP was purified from *E. coli* cells using glutathione-Sepharose 4B beads, and the proteins were reacted with microsomal proteins from Arabidopsis etiolated seedlings. The boundary matrices were subjected to SDS-PAGE and then immunoblot using anti-GST, anti-phot1, and anti-H^+-ATPase antibodies. (**a**) Pull-down of assay of phot1 and BHP. (**b**) Pull-down of assay of H^+-ATPase and BHP. The GST-14-3-3 was used as a positive control for the binding to phot1 and the H^+-ATPase. (**c**) *In vitro* pull-down assay for interaction of BHP with PP1. Both proteins were expressed in *E. coli* and used. Extract from *E. coli* cells expressing GST or GST-BHP was mixed with that expressing FLAG-PP1 and reacted with glutathione-Sepharose 4B beads. Proteins on the beads were subjected to SDS-PAGE and then immunoblotting using anti-GST and anti-FLAG antibodies.

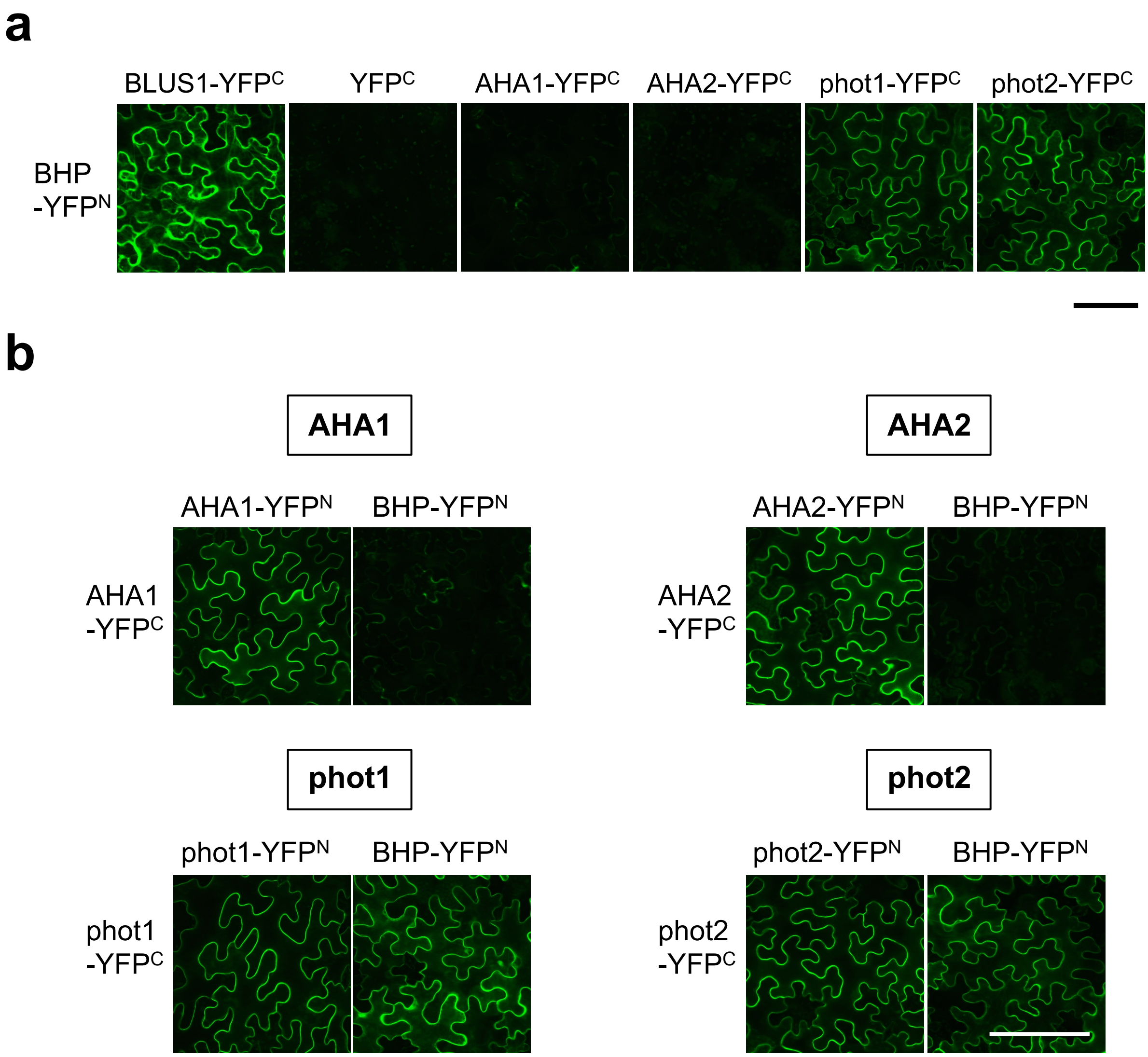

Supplementary Figure 8. *In vivo* interactions of BHP with signaling components in blue light-dependent stomatal opening by BiFC assay.

(**a**) Fluorescence images of the interactions between BHP and BLUS1, AHA1, AHA2, phot1, and phot2 in BiFC assays. Experiments were performed as shown in Fig. 5b. For negative control, the C-terminal half of YFP (YFP^{C}) only was co-expressed with BHP-YFP^{N}. Scale bar is 150 μm. (**b**) Comparison of BHP-H^{+}-ATPase or -phototropin interactions with the positive interaction controls in BiFC assays. Because H^{+}-ATPase and phototropins form multimer complexes[2,3], co-expression of AHA1-YFP^{N}/AHA1-YFP^{C}, AHA2-YFP^{N}/AHA2-YFP^{C}, phot1-YFP^{N}/phot1-YFP^{C}, and phot2-YFP^{N}/phot2-YFP^{C} were used as positive controlsScale bar represents 150 μm.

Supplementary Table 1. Putative targets of the selected inhibitors in Arabidopsis.

Inhibitor	Mammalian target	Putative targets in Arabidopsis	Identity (%)
Tyrphostin 9	PDGFRK	Raf-like kinase At4g31170	39
		Raf-like kinase At2g24360	37
		Raf-like kinase At4g35780	32
Sphingosine	PKC	S6 kinase-1 At3g08730	38
		S6 kinae-2 At3g08720	41
		Putative protein kinase At1g48490	39
GW5074	c-Raf	Raf-like kinase At4g35780	38
		Raf-like kinase At2g17700	38
		Raf-like kinase At1g73660	37
BML-265	EGFRK	Raf-like kinase At4g35780	32
		Raf-like kinase At4g38470	32
		Raf-like kinase At2g17700	33

Tyrphostin 9, Sphingosine, GW5074 and BML-265 were screened from the Inhibitor library (Enzo; BML-2832) as inhibitors that suppress effectively the blue light-dependent H^+-ATPase phosphorylation and stomatal opening. Detection of the guard cell H^+-ATPase was performed by the immunohistochemical method (Fig. 1a). The targets of the inhibitors are known in the mammalian cells. Putative targets in Arabidopsis were assumed by BLAST search (http://blast.ncbi.nlm.nih.gov/Blast.cgi) using the full-length amino acid sequences, and three proteins with high similarity are shown in the list.

Supplementary Table 2. Primers used in RT-PCR and BiFC assay.

Gene	Experiments	Primer sequence
At3g58640<br>B1	for RT-PCR	F: ATGGCGATCAAAGAGGAGAC<br>R:TAGGCCTGAAAAGTAACCAC
At4g24480<br>B3	for RT-PCR	F: TCAGAGTGAGATTTTAACGAAATGC<br>R:CTATAGTATGGGAGCTGATTTAGTTGG
At2g35050<br>B4	for RT-PCR	F: ATGGATCAAGCAAAAGGTTATGAACATG<br>R: TTACTTGTGGATTTGGTGGTTGACAG
At3g24715<br>B4	for RT-PCR	F: ATGTCAGATAGATGGGCTCGAC<br>R: AGGAAATTGGCAATGGGAGAG
VIK At1g14000<br>C1	for RT-PCR	F: CAATGGTGATCTGATGGTAG<br>R: TCTGAATCGGTTTTGAGATG
BHP At4g18950<br>C1	for RT-PCR	F: ATGGAAGAGGATTATCAACAGC<br>R: TCACAAATGTGAACCGGATG
At4g35780<br>C2	for RT-PCR	F: ATGGCGATCAAAGAGGAGAC<br>R: TAGGCCTGAAAAGTAACCAC
At5g50180<br>C3	for RT-PCR	F: AATCTTGCAATGGATTCTTTGACTG<br>R: CAAGGTTAATAACATTGATTGAAGCAG
At2g24360<br>C6	for RT-PCR	F: AGTGAATCTTAGATTGGGAAGATGC<br>R: TTCTCGCTGTCGTCATTATCTCTG
At3g01490<br>C7	for RT-PCR	F: AAATGAAGGAGAAGGCGGAGAGTG<br>R: ATCCCAAAGCTATACACATCGCAC
At5g50000<br>C7	for RT-PCR	F: ATGAAAGAAGGAAAGGATGGGTTTG<br>R: TTAAGGACCACGTTTCCTTCGG
**BHP**<br>BHP-YFP^N	for BiFC	F: GAGGGTACCGCTCCCATGGAAGAGGATTATCAACAGC<br>R: CTTTTGCTCCATCCCCAAATGTGAACCGGATGATG
**BLUS1**<br>BLUS1-YFP^C	for BiFC	F: GAGGGTACCGCTCCCATGGCTCGGAACAAGCTCGAGTTC<br>R: GTATGGGTACATCCCACCCAAAACACTATCTTTATCAGC
**PHOT1**<br>PHOT1-YFP^N	for BiFC	F: GAGGGTACCGCTCCCATGGAACCAACAGAAAAACCATCG<br>R: CTTTTGCTCCATCCCAAAAACATTTGTTTGCAGATCTTCTAGC
PHOT1-YFP^C	for BiFC	F: GAGGGTACCGCTCCCATGGAACCAACAGAAAAACCATCG<br>R: GTATGGGTACATCCCAAAAACATTTGTTTGCAGATCTTCTAGC
**PHOT2**<br>PHOT2-YFP^N	for BiFC	F: GAGGGTACCGCTCCCATGGAGAGGCCAAGAGCCCCT<br>R: CTTTTGCTCCATCCCGAAGAGGTCAATGTCCAAGTCCG
PHOT2-YFP^C	for BiFC	F: GAGGGTACCGCTCCCATGGAGAGGCCAAGAGCCCCT<br>R: GTATGGGTACATCCCGAAGAGGTCAATGTCCAAGTCCG
**AHA1**<br>AHA1-YFP^N	for BiFC	F: GAGGGTACCGCTCCCATGTCAGGTCTCGAAGATATCAAG<br>R: CTTTTGCTCCATCCCCACAGTGTAGTGATGTCCTGC
AHA1-YFP^C	for BiFC	F: GAGGGTACCGCTCCCATGTCAGGTCTCGAAGATATCAAG<br>R: GTATGGGTACATCCCCACAGTGTAGTGATGTCCTGC
**AHA2**<br>AHA2-YFP^N	for BiFC	F: GAGGGTACCGCTCCCATGTCGAGTCTCGAAGATATCAAG<br>R: CTTTTGCTCCATCCCCACAGTGTAGTGACTGGGAGTTTC
AHA2-YFP^C	for BiFC	F: GAGGGTACCGCTCCCATGTCGAGTCTCGAAGATATCAAG<br>R: GTATGGGTACATCCCCACAGTGTAGTGACTGGGAGTTTC
**PP1**<br>TOPP4-YFP^C	for BiFC	F: GAGGGTACCGCTCCCATGGCGACGACGACGACG<br>R: GTATGGGTACATCCCAATCTTTGTGGACATCATGAACTTG
**INHIBITOR3**<br>INHIBITOR3-YFP^N	for BiFC	F: GAGGGTACCGCTCCCATGAGCACAGCAACAAGGCCTTCC<br>R: CTTTTGCTCCATCCCGTCAACGGCTTTAGAATCATTAGAAG

Supplementary References

1. Ichimura, K. *et al*. Mitogen-activated protein kinase cascades in plants: a new nomenclature. *Trends Plant Sci* **7**, 301-308 (2002).

2. Kanczewska, J. *et al*. Activation of the plant plasma membrane H^+-ATPase by phosphorylation and binding of 14-3-3 proteins converts a dimer into a hexamer. *Proc Natl Acad Sci USA* **102**, 11675-11680 (2005).

3. Katsura, H., Zikihara, K., Okajima, K., Yoshihara, S. & Tokutomi, S. Oligomeric structure of LOV domains in Arabidopsis phototropin. *FEBS Lett* **583**, 526-530 (2009).
